# Combinatorial Treatment with Chlorogenic Acid and Cinnamaldehyde Disrupts Intracellular pH and Metabolic Transport in Breast Cancer Cells

**DOI:** 10.3390/molecules31162939

**Published:** 2026-08-21

**Authors:** Yusuff Olayiwola, Vindya Edgunpati, Li Li, Lauren Gollahon

**Affiliations:** 1 Department of Biological Sciences, Texas Tech University, 2500 Broadway, Lubbock, TX 79407, USA; yolayiwo@ttu.edu; 2 North Texas Clinical Pharmacology Cancer Core, 5920 Forest Park Road, Dallas, TX 75235, USAli.li@ttuhsc.edu (L.L.)

**Keywords:** natural products, breast cancer cells, cancer energy metabolism, reversed pH gradient, metabolic transport proteins, first-pass metabolism, intracellular drug uptake, cinnamaldehyde, Akt pathway

## Abstract

Breast cancer cells exhibit a reversed pH gradient and metabolic plasticity that promote proliferation, invasion, and resistance to therapy. Natural products such as chlorogenic acid (CGA) and cinnamaldehyde (CA) have shown emerging anticancer potential. However, their effects on intracellular pH and metabolic transport systems remain undefined. Therefore, the aim of this study was to characterize these parameters in breast cancer and non-tumorigenic breast cells. This study evaluated the physiochemical properties of CGA and CA using LC–MS, under pH conditions (pH 1.2, 7.4, and 9.0) mimicking the gastrointestinal track (GIT). Additionally, LC–MS-based human liver microsome (HLM) assays with NADPH were used to evaluate susceptibility to CYP-mediated metabolism to evaluate first-pass metabolic stability. Intracellular uptake kinetics were quantified at multiple time points using LC–MS. Following CGA:CA treatment, intracellular pH (pH_i_) was measured in cancerous MDA-MB-231 and non-tumorigenic MCF-10A breast cell lines using SNARF-1 targeted ratio-metric fluorescence approach. Expression of OATP1B1, GLUT1, and MCT1 were analyzed by Western and immunofluorescence respectively, to assess potential cellular uptake of CGA:CA through OATP1B1 and their effects on glucose uptake and lactate and proton transport. Physiochemical results demonstrated that the compounds ranged from fully stable (pH 1.2 and 7.4) to completely unstable (pH 9.0). HLM incubation indicated no CYP-mediated hepatic metabolism. Treatment results showed that there was rapid intracellular uptake of CGA and CA in cancer cells and that CGA:CA lowered pH_i_ in both MDA-MB-231 and MCF-7 cells, while pH_i_ remained mostly unchanged in MCF-10A cells. Protein analysis revealed that CGA:CA treatment downregulated GLUT1 and MCT1 expression in cancer cells, suggesting impaired glycolytic activity and lactate shuttling. OATP1B1 expression was significantly suppressed in cancer cells, suggesting feedback inhibition of the solute carrier protein. Collectively, these findings indicate that CGA and CA exhibit favorable biochemical stability and disrupt intracellular pH regulation and metabolic transporter expression in breast cancer cells. Importantly, normal cells are not significantly affected. Thus, CGA:CA demonstrates therapeutic potential for breast cancer through pH_i_ and metabolic modulation.

## 1. Introduction

Breast cancer remains a leading cause of cancer-related mortality and morbidity worldwide. While survival rates for early-stage diseases are high, advanced, metastatic, and triple-negative breast cancer (TNBC) subtypes often display significant treatment resistance and relapse [1,2,3]. Breast cancer progression is driven by a complex interplay of metabolic reprogramming, altered intracellular pH regulation, and dysregulated signaling pathways that collectively support cell proliferation, survival, and metastasis [1,2,3]. A hallmark of malignant cells is the maintenance of a reversed pH gradient—characterized by an abnormally alkaline intracellular environment and an acidic extracellular space—which promotes glycolytic flux, enhances invasive behavior, and stabilizes oncogenic signaling networks [3,4,5]. This phenotype is sustained by the overexpression of key metabolic transport proteins, including glucose transporter 1 (GLUT1), monocarboxylate transporter 1 (MCT1), and the organic anion-transporting polypeptide OATP1B1 [6,7,8,9,10,11,12,13]. Together, these transporters facilitate high glucose uptake, efficient lactate export, and increased import of anionic metabolites, enabling cancer cells to maintain metabolic flexibility and resist stress [6,7,8,9,10,11,12,13].

Natural products have emerged as promising modulators of cancer metabolism due to their multi-targeted mechanisms and low toxicity profiles [14,15,16,17,18]. Chlorogenic acid (CGA), a polyphenolic compound abundant in coffee and plant-derived foods, exhibits antioxidant, anti-neoplastic, anti-inflammatory, and metabolic-regulatory properties [14,15,19,20,21]. Cinnamaldehyde (CA), the major bioactive component of cinnamon, has been shown to modulate redox balance, disrupt mitochondrial function, and interfere with cancer cell survival pathways [14,15,22]. Recent evidence suggests that combining natural compounds with complementary biochemical properties may enhance anticancer efficacy through synergistic disruption of metabolic and signaling networks [15,18,23,24,25,26,27,28]. Although the phytochemicals are structurally different, their complementary biochemical properties may account for their synergistic antineoplastic effects. CGA is a hydrophilic polyphenolic ester composed of caffeic acid and quinic acid, rich in hydroxyl groups and functioning primarily as an antioxidant that modulates AMPK activity, glycolytic flux, and lactate transport. CA, conversely, is a moderately lipophilic phenylpropanoid aldehyde containing an electrophilic α,β unsaturated carbonyl that can react with, and modify, thiol-containing proteins, elevates intracellular ROS, and disrupts Akt and NF κB signaling [15,18,23,24,25,26,27,28]. Despite these differences, both compounds can exploit key cancer vulnerabilities. Namely, they suppress PI3K/Akt signaling, interfere with glycolysis and the pH regulating machinery, and destabilize mitochondrial function. Their synergy may be driven by their ability to reach intracellular compartments to engage various molecular targets and exert different, yet complementary, redox pressures, ultimately producing a coordinated collapse of metabolic plasticity and survival signaling in cancer cells [15,18,23,24,25,26,27,28]. However, the mechanistic basis for such synergy, particularly in the context of metabolic transport, intracellular pH regulation, and cellular uptake, remains insufficiently understood.

Before therapeutic development can commence, it is essential to characterize the biochemical stability and metabolic fate of candidate compounds [29,30,31]. Gastrointestinal pH stability determines oral viability, while human liver microsome (HLM) assays provide insight into susceptibility to cytochrome P450 (CYP)-mediated metabolism [32,33]. Additionally, intracellular uptake kinetics influence bioactivity, especially for compounds whose mechanisms depend on intracellular accumulation [16]. For CGA and CA, which differ in polarity, acidity, and membrane permeability, understanding their combined uptake behavior is critical for interpreting downstream cellular effects [29,34,35], alongside their pharmacodynamic behaviors, such as intracellular pH modulation and the regulation of metabolism and metabolic transport proteins.

This study built upon our previously reported findings [14,21], investigated the biochemical stability, first-pass hepatic metabolism, intracellular uptake, and functional cellular effects of a CGA and CA combination (CGA:CA), in a breast cancer cell model. We assessed compound stability under simulated gastrointestinal (GIT) pH conditions (pH 1.2, 7.4, and 9.0) and evaluated CYP-dependent metabolism using HLM assays with NADPH coenzyme. Intracellular uptake kinetics were quantified by LC–MS across multiple time points to determine the rate and selectivity of CGA and CA accumulation in cancer versus non-cancerous cells. We further examined the impact of CGA:CA on intracellular pH using a SNARF-1 ratio-metric fluorescence approach and evaluated changes in key metabolic transport proteins (GLUT1, MCT1, OATP1B1) using immunofluorescence and Western blotting.

**Figure 1:**
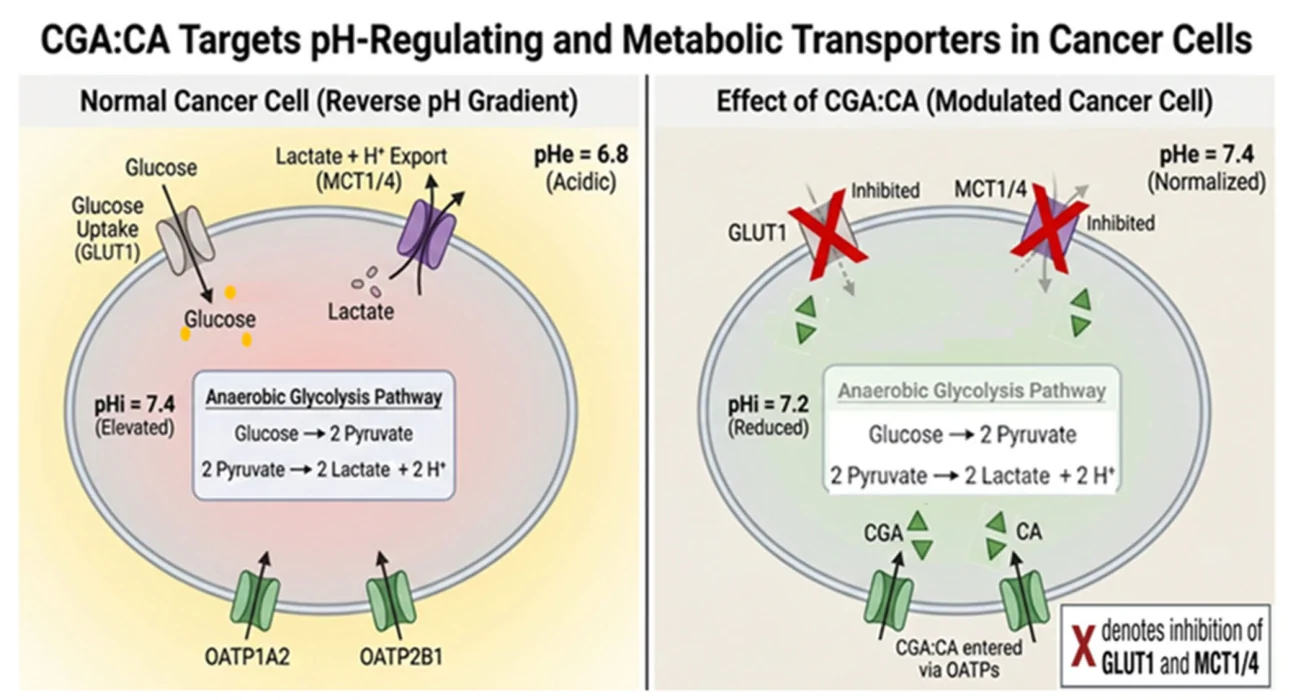
Hypothetical mechanism of action of CGA:CA towards modulating intracellular pH of cancer cells. The left panel illustrates a cancer cell exhibiting the characteristic reversed pH gradient, with elevated intracellular pH (pH_i_ ∼7.4) and acidic extracellular pH (pH_e_ ∼6.8), driven by enhanced glucose uptake via GLUT1 and efficient lactate/H^+^ export through monocarboxylate transporters (MCT1/4). The right panel depicts the effect of combinatorial chlorogenic acid and cinnamaldehyde (CGA:CA) treatment, showing reduced GLUT1-mediated glucose uptake and inhibition of MCT1/4-dependent lactate and proton export. CGA and CA enter cancer cells via organic anion transporting polypeptides (OATP), leading to reduced glycolytic flux, intracellular proton accumulation, and a decrease in pH_i_ (∼7.2), thereby disrupting the cancer-specific reversed pH gradient, and alkaline pH-dependent survival mechanisms in cancer cells.

We hypothesized that the CGA:CA combination negatively regulating Akt signaling globally in cancer cells [14] acts to disrupt the metabolic transporter expression and concomitantly destabilizes the pH homeostasis in breast cancer cells selectively (Figure 1). Because cancer cells overexpress OATP1B1, CGA and CA will enter malignant cells more readily than non-tumorigenic cells, resulting in higher intracellular exposure. Once inside, the combination would directly suppress GLUT1-mediated glucose uptake and MCT1-dependent lactate/H^+^ export, while indirectly reducing glycolytic proton production by limiting substrate availability. These combined effects will restrict both the generation and removal of acid equivalents, causing intracellular proton accumulation and collapse of the cancer-specific reversed pH gradient. Non-cancerous cells, with lower transporter expression and a more resilient pH-regulatory capacity, will maintain relatively stable intracellular pH. Thus, CGA:CA will exploit transporter overexpression and fragile pH regulation in cancer cells to induce selective intracellular acidification and functional impairment (summarized in Figure 1).

Collectively, this study provides a more comprehensive mechanistic framework describing how the CGA:CA combination interacts with cancer cell metabolism, pH regulation, and transporter expression. Our findings reveal that CGA and CA are biochemically stable, bypass CYP-mediated metabolism, accumulate rapidly and selectively in cancer cells, and disrupts metabolic transport and pH homeostasis, highlighting their potential as a low-toxicity, metabolic-signaling modulator for breast cancer therapy.

## 2. Materials and Methods

### 2.1. Cell Lines and Cell Culture

The breast cancer cell lines MDA-MB-231 and MCF-7 cells, and normal mammary cells MCF-10A were used to investigate our hypothesis. The MDA-MB-231 cell line (ATCC, catalog# HT-26, ATCC, Gaithersburg, MD, USA) is an epithelial, human breast cancer cell line with high metastatic capacity. It is a highly aggressive and poorly differentiated triple-negative breast cancer (TNBC) cell line as it lacks both estrogen and progesterone receptors, as well as HER2 (human epidermal growth factor receptor 2) [36]. The MCF-7 cell line (ATCC, catalog# HT-22, ATCC, Gaithersburg, MD, USA), is a human breast cancer cell line with estrogen, progesterone, and glucocorticoid receptors [21,36]. They are poorly aggressive, have low invasive potential, and have been widely used for various cancer studies, including therapeutic research. The MCF-10A cell line is a non-tumorigenic, immortalized human mammary epithelial cell line derived from fibrocystic breast tissue and is characterized by a near-diploid karyotype, rapid proliferation, and ability to form 3D acinar structures [21,36]. The breast cancer and normal cell lines were cultured in high-glucose Dulbecco’s Modified Eagle’s Medium and RPMI 1640 medium (DMEM; Gibco, Waltham, MA, USA) supplemented with 10% fetal bovine serum (FBS; ATCC, Rockville, MD, USA) and 1% penicillin/streptomycin in a humidified incubation atmosphere of 5% CO_2_ at 37 °C, respectively.

### 2.2. Cell Treatments

Chlorogenic acid (CGA) was purchased from Thermo Fisher (Thermo Fisher Scientific, Catalog #J60457.MD; Waltham, MA, USA) (≥95% purity) in powder form and was dissolved in PBS to a stock concentration of 300 μg/mL. Cinnamaldehyde (CA) was obtained from Thermo Fisher (Thermo Fisher Scientific, Catalog #A14689.36; Waltham, MA, USA) (98+% purity) in liquid form. The stock concentration was 7.9 Mm and was aliquoted for use. Stock solutions were stored at room temperature according to the manufacturer’s guidelines for further use. In our previously reported studies [21,36], the half-maximal inhibitory concentration (IC_50_) of both CA and CGA were determined in both MDA-MB-231 and MCF-7 cells. The IC_50_ of CA in both MDA-MB-231 and MCF-7 cells were 42.5 µM and 35 µM, respectively, while the IC_50_ of CGA in both MDA-MB-231 and MCF-7 cells were 225 µg/mol and 250 µg/mol, respectively. A mixture of CGA and CA was used in this study to treat both cancer and non-cancerous breast cells. For Western blotting analysis in this study, CGA and CA were combined and applied to MDA-MB-231, MCF-7, and MCF-10A breast cell lines at three concentration levels (Conc 1, 2 and 3) to evaluate dose-dependent effects. For MDA-MB-231 cells, the treatment combinations consisted of Conc1 at 25 µg/mL CGA + 10 µM CA, Conc2 at 50 µg/mL CGA + 20 µM CA, and Conc3 at 100 µg/mL CGA + 30 µM CA. For MCF-7 and MCF-10A cells, the corresponding combinations were Conc1 at 25 µg/mL CGA + 10 µM CA (which was the same as in MDA-MB-231 cells, Conc2 at 50 µg/mL CGA + 15 µM CA, (which differed in CA from MDA-MB-231 cells), and Conc3 at 100 µg/mL CGA + 20 µM CA (which also differed in CA from the MDA-MB-231 cells). These paired concentrations were selected to ensure comparable exposure ranges across both cell lines and to assess potential synergistic interactions between CGA and CA at increasing doses.

### 2.3. LC–MS-Based Assessment of the Impact of GIT-Equivalent pH on the Stability of CGA and CA

The stability of chlorogenic acid (CGA) and cinnamaldehyde (CA) was assessed by incubating each compound (CGA: 20 µg/mL and CA: 5 µM) at 37 °C in buffer solutions adjusted to pH 1.2, 7.4, and 9.0, using HCl and NaOH, to represent physiologically relevant gastric and intestinal pH environments. Samples were collected at 0, 30, 60, 180, and 360 min. Following incubation, all samples were stored at −80 °C until analysis. On the day of analysis, samples were thawed, vortex-mixed thoroughly, and centrifuged at 10,000× *g* rpm for 10 min at 4 °C to remove particulates. A 1 µL aliquot of supernatant was used for LC–MS analysis [37].

Analyses were conducted using a UPLC system (Shimadzu Nexera, Shimadzu Corporation, Kyoto, Japan) equipped with a Luna C18 column (3 µm, 150 × 2 mm, 100 Å). Mobile phases consisted of water with 0.1% formic acid (A) and acetonitrile with 0.1% formic acid (B), operated at a flow rate of 0.4 mL/min with a linear gradient of 5% B (0–1 min), increasing to 95% B by 6.5 min, held until 8 min, and re-equilibrated to 5% B by 10 min. Mass spectrometric detection was performed on a Sciex ZenoTOF 7600 (SCIEX, Framingham, MA, USA) operated in negative TOF-MS mode (*m*/*z* 100–1000). Data were processed in Sciex OS software (version 3.0). CGA was quantified using the deprotonated molecular ion at *m*/*z* 353 (RT 3.63 min). CA was monitored through its oxidized product ion at *m*/*z* 147 (RT 5.11 min), as the parent aldehyde ion (*m*/*z* 131) was not detectable under the applied analytical conditions. Peak areas at *m*/*z* 353 and *m*/*z* 147 were used to evaluate degradation across pH conditions and time points.

### 2.4. LC–MS-Based Assessment of Human Liver Microsomal Metabolism of CGA and CA

The metabolic stability of chlorogenic acid (CGA) and cinnamaldehyde (CA) was evaluated using pooled human liver microsomes (HLM) (Thermo Fisher Scientific, Catalog#: HMMCPL; Waltham, MA, USA) (Purity: Research Grade) to assess susceptibility to cytochrome P450 (CYP)-mediated Phase I metabolism. Incubations were performed under two conditions: HLM supplemented with NADPH (Thermo Fisher Scientific, Catalog# 044126.MD; Waltham, MA, USA), the essential cofactor for CYP-dependent oxidative reactions, and HLM incubated without NADPH to distinguish enzymatic metabolism from non-CYP-mediated degradation [32]. To assess whether degradation was concentration-dependent and to evaluate reaction kinetics, CGA was incubated at 25 µg/mL and 50 µg/mL, while CA was incubated at 10 µM and 20 µM. Incubation samples were collected at 0, 30, and 60 min to monitor time-dependent loss of parent compounds and potential formation of metabolites. Following incubation, samples were stored at −80 °C until analysis. On the day of analysis, samples were thawed, vortex-mixed, and centrifuged at 10,000× *g* rpm for 10 min at 4 °C. Supernatants (5 µL for CGA and 20 µL for CA) were injected for LC–MS analysis. Quantification was performed using integrated peak areas corresponding to CGA (*m*/*z* 353.0818, RT ≈ 3.64 min) and the stable oxidized product of CA (cinnamic acid; *m*/*z* 147.0427, RT ≈ 5.17 min), as the parent aldehyde ion (*m*/*z* 131) was not detectable under the applied analytical conditions. Data were processed in Sciex OS software. Metabolic stability was assessed by comparing parent compound abundance over time in the presence and absence of NADPH. The absence of time-dependent parent loss, concentration-dependent depletion, or additional metabolite formation was interpreted as a lack of CYP-mediated hepatic metabolism under the conditions tested.

### 2.5. Intracellular Drug Uptake Study

The intracellular uptake of chlorogenic acid (CGA) and cinnamaldehyde (CA) was evaluated in MDA-MB-231 breast cancer cells and MCF-10A control breast epithelial cells to assess differences in intracellular accumulation between non-cancerous and cancerous cell types. Cells (seeding density of 1.2 × 10^6^/mL) were treated with combination of CGA (25 μg/mL) and CA (10 μM) in 12-well plates and intracellular levels were quantified over multiple time points (0, 30, 60 and 120 min) using LC–MS to determine uptake kinetics and retention profiles [38]. Following treatment, cells were harvested and pelleted, and intracellular compounds were extracted using a lysis buffer consisting of 0.1% Triton X-100 (70 µL), dimethyl sulfoxide (50 µL), and butylated hydroxytoluene (BHT; 5 µL, prepared at 90 mg/mL in DMSO) to prevent oxidative degradation. Samples were vortexed briefly and incubated at room temperature for 20 min to ensure complete cell lysis. Methanol (125 µL) was then added to precipitate proteins, followed by shaking for 20 min. Lysates were centrifuged at 10,000× *g* rpm for 10 min at 4 °C, and the resulting supernatants were collected for analysis, with 20 µL injected per sample.

Quantification of intracellular CGA and the oxidized form of CA was performed using LC–MS with calibration curves prepared in the same biological matrix as the samples. Standard curves covered a concentration range of 0.1–1000 ng/mL and included four quality control levels (300, 30, 3, and 0.3 ng/mL) to ensure assay accuracy and precision. Mass spectrometric detection was carried out in MRM-HR mode using a ZenoTOF 7600 system, monitoring the transitions *m*/*z* 353.0819 → 191.0535 for CGA and *m*/*z* 147.0436 → 103.0542 for oxidized CA. Data acquisition and quantitation were performed using Sciex OS software. Intracellular concentrations were normalized to total cellular protein content, (quantified from parallel wells using a BCA assay), and compared between MCF-10A and MDA-MB-231 cells across time points to evaluate differential uptake and retention of CGA and CA in malignant versus non-malignant breast epithelial cells.

### 2.6. Intracellular pH Measurements Performed Using a pH-Sensitive Fluoroprobe with a Ratio-Metric Fluorescence Approach

Intracellular pH (pH_i_) was quantified using the pH-sensitive fluoroprobe SNARF-1-AM (Thermo Fisher Scientific, Catalog# S23921; Waltham, MA, USA) and a ratio-metric fluorescence approach. MDA-MB-231 and MCF-7 breast cancer cells, as well as non-tumorigenic MCF-10A breast epithelial cells, were cultured under standard conditions and treated with the CGA:CA combination (50 μg/mL of CGA and 20 μM of CA ratio 1:1) for defined time points (0, 0.5, 1, and 2 h). Following treatment, cells were washed with phosphate-buffered saline (PBS) and incubated with SNARF-1-AM prepared in serum-free medium supplemented with Pluronic F-127 (CGA) (Thermo Fisher Scientific, Catalog# P3000MP; Waltham, MA, USA) (≥95% purity) to facilitate probe solubilization and cellular loading [39]. Cells were loaded for 20 min at 37 °C to allow probe uptake and intracellular ester hydrolysis. After loading, cells were washed, harvested, and resuspended in fresh medium. Fluorescence emission was measured using a fluorescence spectrophotometer, Bio-Tek Synergy H1 Microplate Reader H1M (Bio-Tek; Winooski, VT, USA), at dual emission wavelengths of approximately 580 nm and 640 nm following excitation at 488 nm. The ratio of fluorescence intensities (580/640 nm) was used as a quantitative measure of intracellular pH, minimizing probe concentration- and photobleaching-related variability.

Calibration curves were generated by incubating SNARF-loaded cells in high-potassium buffers adjusted to defined pH values (5.0–9.0) and containing nigericin (Thermo Fisher Scientific, Catalog# N1495; Waltham, MA, USA) to equilibrate intracellular and extracellular pH. Fluorescence ratios were plotted against known pH values and fitted to a calibration function, which was subsequently used to calculate intracellular pH values in CGA- and CA-treated samples. Intracellular pH measurements were compared across cell types and time points to assess differential pH_i_ modulation in cancerous versus non-cancerous breast epithelial cells.

### 2.7. Analysis of Transporter Expression by Immunofluorescence

Transporter expression was assessed by immunofluorescence staining for GLUT1, MCT1, and OATP1B1. Cells were seeded onto sterile 8-well chamber slides and allowed to adhere overnight. Following treatment with CGA:CA for 6 h, with only one combinatorial concentration—25 μg/mL of CGA and 10 μM of CA ratio 1:1, using culture media of 300 μL/well—cells were washed twice with cold PBS and fixed in 4% paraformaldehyde for 15 min at room temperature. Fixed cells were rinsed with PBS and permeabilized using 0.1% Triton X-100 in PBS for 10 min, followed by blocking in 5% bovine serum albumin (BSA) for 1 h to reduce nonspecific binding [21]. Cells were incubated for 1 h at room temperature with rabbit-hosted primary antibodies: anti-GLUT1 (Product# 73015S, Cell Signaling Technology, Danvers, MA, USA), anti-MCT1 (Product# 76508S, Cell Signaling Technology, Danvers, MA, USA), and anti-OATP1B1 (Thermo Fisher Scientific, Catalog# PA5-106753; Waltham, MA, USA), diluted in ratios of 1:200, 1:500, and 1:500 respectively, with the blocking buffer as a diluent. After primary incubation, cells were washed three times with PBS and incubated for 1 h at room temperature with goat anti-rabbit FITC-conjugated secondary antibody (Thermo Fisher Scientific, Catalog# 65-6111; Waltham, MA, USA), diluted in ratio 1:2000 with the blocking buffer as a diluent. Nuclei were counterstained with DAPI for 5 min, and coverslips were mounted onto glass slides using an antifade mounting medium.

Fluorescence images were acquired using an Echo Revolve 4-in-1 microscope (Discover Echo Inc., San Diego, CA, USA, 92126) equipped with appropriate filter sets. Exposure settings were kept constant across all groups for each transporter. Image analysis was performed in ImageJ (version 1.54g) by quantifying mean fluorescence intensity per cell or per field, normalized to background signal. Transporter expression levels were compared between control and treated groups to assess CGA:CA-induced changes in GLUT1, MCT1, and OATP1B1 abundance.

### 2.8. Western Blot Analysis of Transporter Expression

Protein expression levels of GLUT1, MCT1, and OATP1B1 were evaluated by Western blotting. Cells were harvested on ice and lysed in RIPA buffer supplemented with protease and phosphatase inhibitors. Lysates were incubated on ice for 20 min with intermittent vortexing and clarified by centrifugation at 14,000× *g* rpm for 15 min at 4 °C to collect supernatants. Protein concentrations were determined using the BCA assay. Volume equivalent of 40 µg of protein was mixed with 6× Laemmli sample buffer containing β-mercaptoethanol (ratio 5:1). The mixture was heated at 95 °C for 5 min before separation on SDS-polyacrylamide gels. Proteins were transferred onto PVDF membranes using a wet transfer system and blocked for 1 h at room temperature in 5% BSA in TBST [31].

Membranes were incubated for 1 h at room temperature with rabbit-hosted primary antibodies diluted in blocking buffer: anti-GLUT1 (Product# 73015S, Cell Signaling Technology, Danvers, MA, USA), anti-MCT1 (Product# 76508S, Cell Signaling Technology, Danvers, MA, USA), and anti-OATP1B1 (Thermo Fisher Scientific, Catalog# PA5-106753; Waltham, MA, USA), at an antibody:diluent dilution ratio of 1:1000, 1:1000, and 1:2000 respectively. After three washes in TBST, membranes were incubated with goat anti-rabbit HRP-conjugated secondary antibody (Product# 7074, Cell Signaling Technology) for 1 h at room temperature, at a 1:2000 dilution ratio. Protein bands were visualized using enhanced chemiluminescence (Thermo Scientific, Catalog# 34577, Waltham, MA, USA) and imaged on a LiCor imaging system (Odyssey^®^ Fc) by LI-COR Biosciences (Lincoln, NE, USA). Densitometry was performed in ImageJ (version 1.54g). Transporter expression was normalized to loading control HSP-60 (Product# 4869S, Cell Signaling Technology, Danvers, MA, USA), and relative expression levels were compared between control and CGA:CA-treated samples.

### 2.9. Statistical Analysis

All statistical analyses were performed using GraphPad Prism (version 10.2.3) and R (version 4.5.3). Unless otherwise stated, all experiments were conducted using three independent biological replicates, each measured in technical triplicate. Data are presented as mean ± SEM, and significance was defined as *p* ≤ 0.05. Prior to applying parametric tests, data distributions were evaluated using the Shapiro–Wilk normality test. Parametric analyses were used only when normality assumptions were met. For simulated GIT pH stability experiments and intracellular pH modulation assays, differences across pH conditions or incubation times were analyzed in GraphPad Prism using one-way ANOVA followed by Tukey’s multiple-comparison test. Microsomal metabolism assays comparing NADPH-dependent and NADPH-independent incubations across time were also evaluated in Prism using one-way ANOVA with Tukey’s correction. Immunofluorescence intensity measurements were compared between control and treatment groups using unpaired two-sample t-tests. Western blot densitometry involving multiple treatment groups was analyzed using one-way ANOVA with Tukey’s HSD post hoc test. For CGA and CA uptake kinetics, unpaired two-sample t-tests were performed at each time point to compare uptake between MDA-MB-231 and MCF-10A cells, and only statistically significant differences were annotated in figures. All R-based visualizations were generated using ggplot2 with custom publication-quality themes.

## 3. Results

### 3.1. Simulated GIT-Equivalent pH Stability of CGA and CA

**Figure 2:**
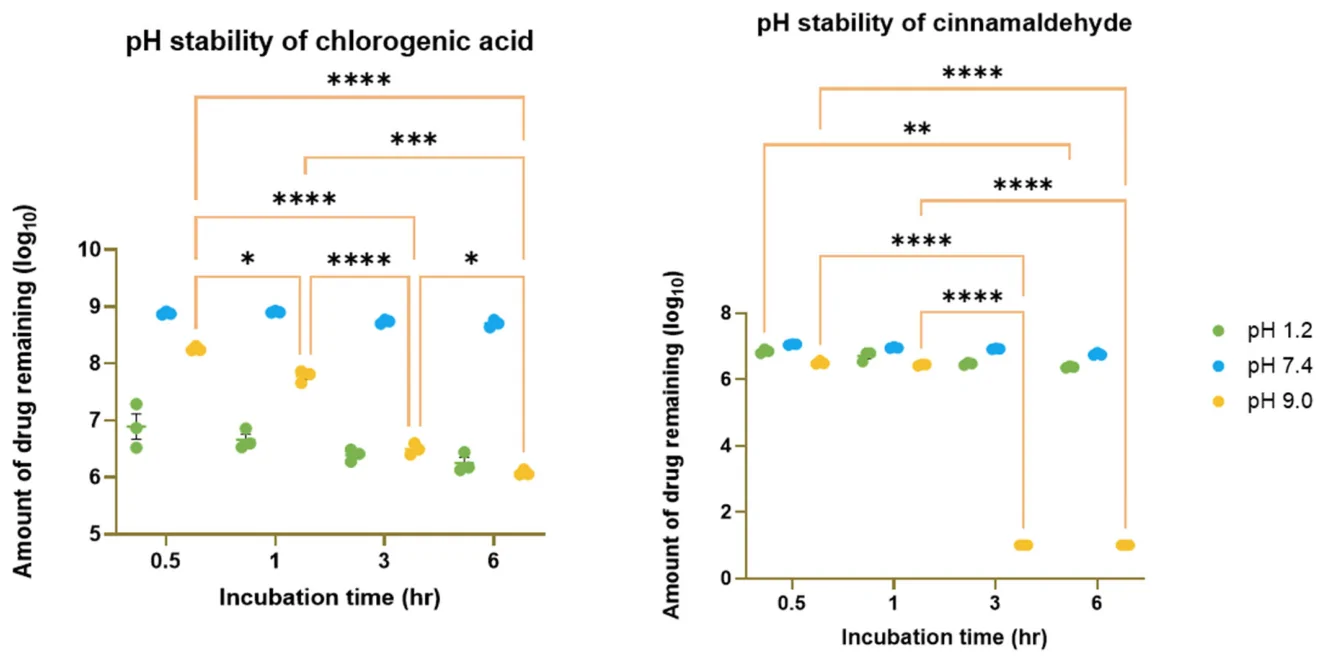
pH-dependent stability of chlorogenic acid (CGA) and cinnamaldehyde (CA) assessed by LC–MS. CGA (**left**) and CA (**right**) were incubated at gastrointestinal-equivalent pH conditions (pH 1.2, 7.4, and 9.0) for up to 6 h, and the remaining compound was quantified by LC–MS. CGA remained stable at pH 1.2 and 7.4, with significant degradation observed at pH 9.0 beginning at 1–3 h. CA showed a similar pattern, with minimal loss at pH 1.2 and 7.4 but marked degradation at pH 9.0 over time. Data are presented as log_10_-transformed peak areas (mean ± SEM). Statistical significance was determined by two-way ANOVA with post hoc multiple-comparison testing. * *p* < 0.05, ** *p* < 0.01, *** *p* < 0.001, and **** *p* < 0.0001 versus pH-matched 0 h control.

The stability of chlorogenic acid (CGA) and cinnamaldehyde (CA) was evaluated across physiologically relevant gastrointestinal pH conditions (pH 1.2, 7.4, and 9.0), using LC–MS quantification of their characteristic ions. As shown in Figure 2, CGA remained highly stable at both gastric (pH 1.2) and physiological (pH 7.4) conditions, with minimal loss in peak area over the 6 h incubation period. In contrast, CGA exhibited marked degradation at alkaline pH 9.0, showing a progressive decline in signal intensity beginning at 60 min and continuing through 360 min, consistent with base-catalyzed hydrolysis of ester-containing phenolic acids. CA exhibits similar degradation patterns as CGA. Although the parent aldehyde (CA, *m*/*z* 131) was not detectable under negative mode TOF MS conditions, CA was reliably quantified via its oxidized product, cinnamic acid, at *m*/*z* 147, which formed consistently during ionization. The *m*/*z* 147 peak remained stable at pH 1.2 and 7.4, indicating minimal degradation under acidic and physiological pH. However, a substantial reduction in the *m*/*z* 147 signal was observed at pH 9.0, reflecting accelerated degradation of CA at alkaline pH. The parallel loss of CGA and CA at pH 9.0 suggests that both compounds are chemically vulnerable under alkaline conditions but remain intact under physiologically relevant gastric and intestinal pH.

### 3.2. Human Liver Microsomal Metabolism of CGA and CA

**Figure 3:**
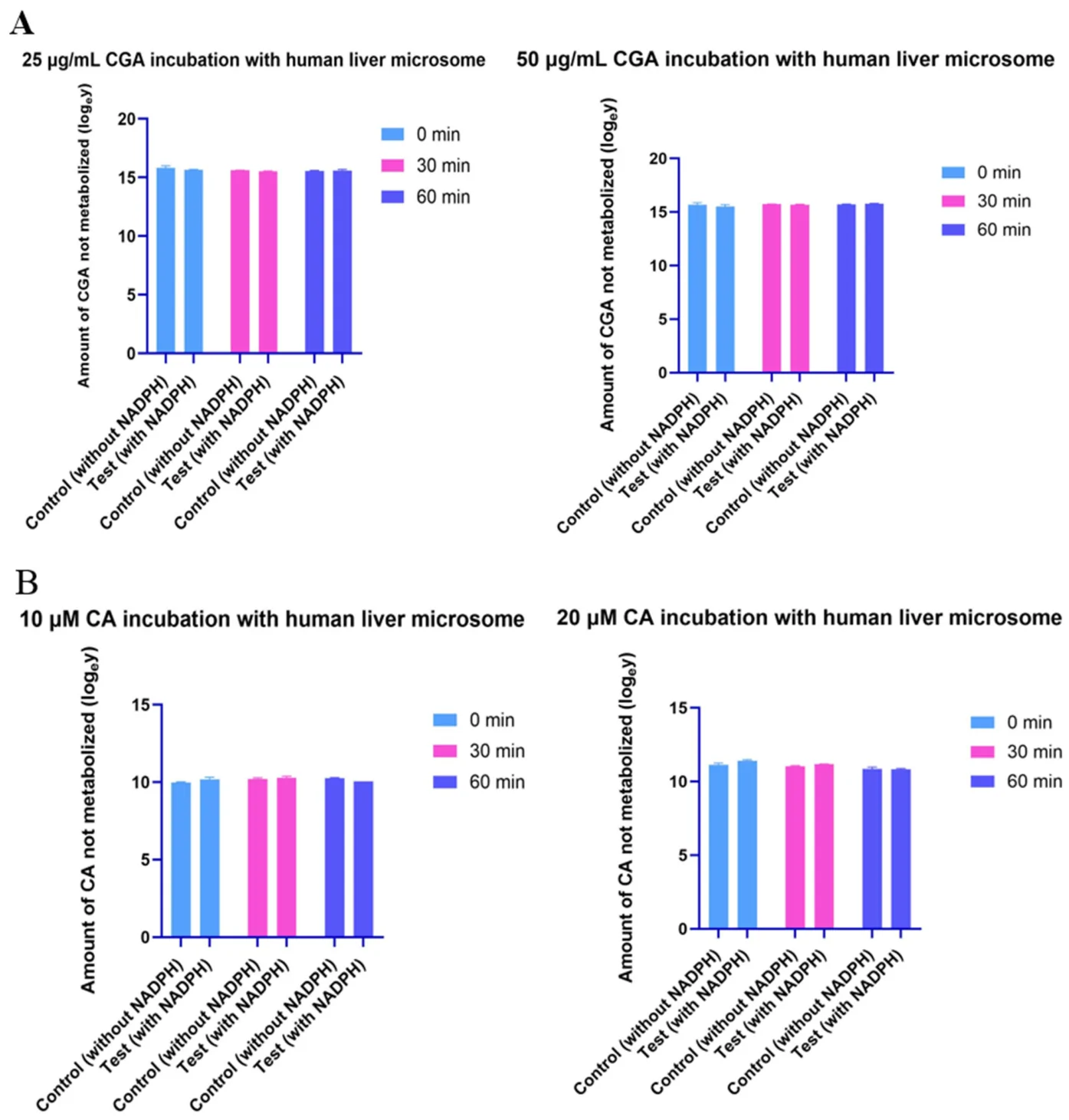
LC–MS-based pooled human microsomal assessment of CGA and CA metabolism by cytochrome P-450. CGA (25 and 50 µg/mL) (**A**) and CA (10 and 20 µM) (**B**) were incubated with pooled human liver microsomes (HLM) under NADPH-supplemented (+NADPH) and NADPH-free (−NADPH) conditions to evaluate potential CYP-mediated Phase I metabolism. LC–MS quantification of CGA (*m*/*z* 353) and CA (monitored via its stable oxidized product at *m*/*z* 147) showed no significant decline in compound abundance over 60 min at any concentration tested. For both CGA and CA, +NADPH and −NADPH profiles were indistinguishable, indicating an absence of NADPH-dependent microsomal metabolism. Bars represent mean ± SEM. Statistical analysis was performed using two-way ANOVA with post hoc multiple comparisons; no statistically significant differences were observed across time points or between +NADPH and −NADPH conditions.

The metabolic stability of chlorogenic acid (CGA) and cinnamaldehyde (CA) was assessed in pooled human liver microsomes (HLM) using two concentrations for each compound to evaluate potential concentration-dependent metabolism (CGA: 25 and 50 µg/mL; and CA: 10 and 20 µM). LC–MS analysis showed that CGA remained highly stable at both concentrations, with no measurable decline in the *m*/*z* 353 peak area over the 60 min incubation period. Importantly, CGA exhibited identical stability profiles in the presence and absence of NADPH, indicating a lack of cytochrome P450-mediated metabolism. CA demonstrated a similar pattern. Because the parent aldehyde (*m*/*z* 131) was not detectable under negative-mode TOF-MS conditions, CA abundance was quantified using its stable oxidized product, cinnamic acid (*m*/*z* 147). The *m*/*z* 147 signal remained unchanged across all time points and was unaffected by NADPH supplementation at both 10 and 20 µM. The absence of concentration-dependent loss and the identical +NADPH/−NADPH profiles confirm that CA is not a substrate for hepatic CYP enzymes under the conditions tested. Together, these findings demonstrate that both CGA and CA exhibit metabolic stability in human liver microsomes across multiple concentrations (Figure 3), with no evidence of NADPH-dependent Phase I metabolism. This suggests that first-pass hepatic clearance is unlikely to limit systemic exposure to either compound following oral administration.

### 3.3. Intracellular Uptake of CGA and CA in MDA-MB-231 and MCF-10A Cells

The intracellular accumulation of chlorogenic acid (CGA) and cinnamaldehyde (CA) was quantified in MDA-MB-231 breast cancer cells and non-tumorigenic MCF-10A breast epithelial cells over a 2 h incubation period. Both compounds exhibited rapid uptake kinetics, with intracellular concentrations peaking at 0.5 h before declining over time.

CGA uptake increased over time in both MDA-MB-231 and MCF-10A cells, with intracellular levels rising sharply from baseline and reaching maximal accumulation at 30 min in MDA-MB-231 cells (~55 ng/mL). MCF-10A cells showed a similar early rise but with a lower peak (~45 ng/mL). After 30 min, CGA levels declined in both cell lines, with MCF-10A exhibiting a more pronounced decrease by 120 min. A statistically significant difference between the two cell lines was observed at the 120 min time point (* *p* < 0.05).

CA displayed a similar rapid uptake phase in both cell lines, peaking at 30 min, with MDA-MB-231 cells reaching higher intracellular concentrations (~38 ng/mL) compared to MCF-10A cells (~30 ng/mL). Following this peak, CA levels progressively declined, again with a steeper reduction in MCF-10A cells by 120 min. Significant differences were detected between baseline and 30 min (*p* < 0.05) and between the two cell lines at 120 min (** *p* < 0.001), indicating differential retention or clearance kinetics.

**Figure 4:**
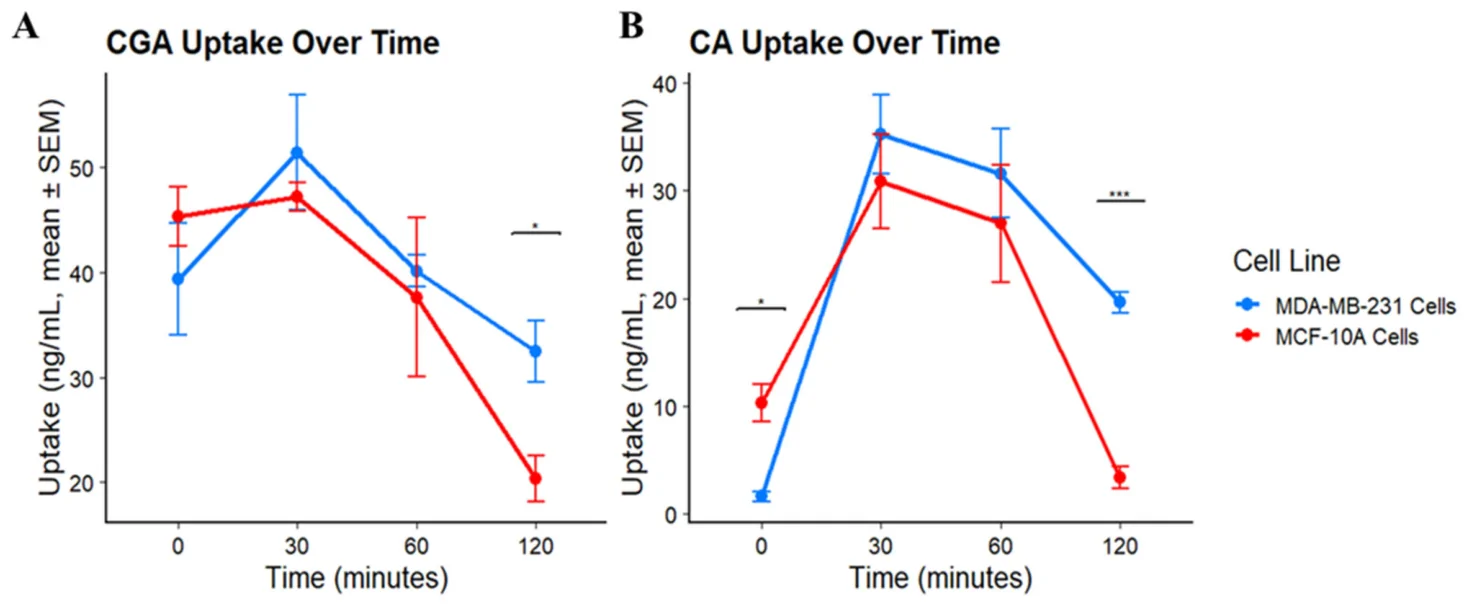
Intracellular uptake kinetics of chlorogenic acid (CGA) and cinnamaldehyde (CA) in MDA-MB-231 and MCF-10A cells. CGA (**A**) and CA (**B**) uptake were quantified in MDA-MB-231 breast cancer cells and non-tumorigenic MCF-10A breast epithelial cells over a 2 h incubation period. Both compounds exhibited rapid uptake, with intracellular concentrations peaking at 0.5 h before declining. CGA and CA demonstrated preferential accumulation in MDA-MB-231 cells at the 0.5 h peak. Data represent mean ± SEM. Statistical significance was assessed by two-way ANOVA with post hoc multiple comparisons. * *p* < 0.05 and *** *p* < 0.001 versus time-matched MCF-10A or baseline control, as indicated.

Together, these uptake profiles demonstrate that both CGA and CA enter cells rapidly but are retained more efficiently in MDA-MB-231 than in MCF-10A cells, highlighting a clear difference in time-dependent intracellular handling between malignant and nonmalignant breast epithelial lines (Figure 4). These differential uptake patterns support the hypothesis that CGA and CA accumulate in cancer cells, potentially contributing to their increased effects on biological activity.

### 3.4. Intracellular pH Modulation by CGA and CA in Breast Cancer and Non-Cancer Cells

**Figure 5:**
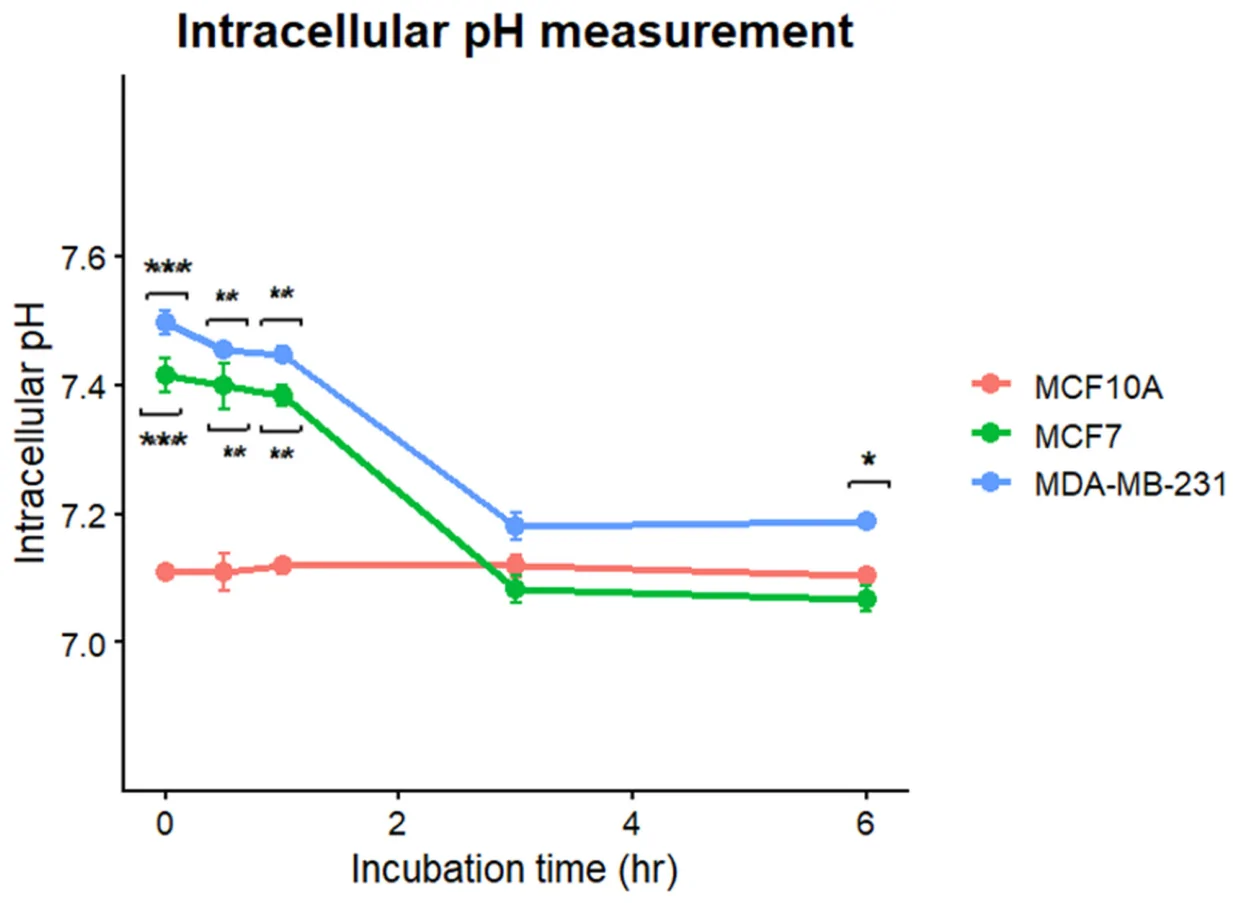
CGA and CA-induced modulation of intracellular pH in breast cancer and non-cancer cells. Intracellular pH (pH_i_) was measured in MDA-MB-231, MCF-7, and non-tumorigenic MCF-10A breast epithelial cells over a 6 h incubation period using a pH-sensitive ratio-metric fluoroprobe. Both MDA-MB-231 and MCF-7 cancer cells exhibited a time-dependent decrease in pH_i_, with acidification becoming evident after 1 h and continuing through 3–6 h. In contrast, MCF-10A cells maintained a stable pH_i_ throughout the experiment, showing minimal deviation from baseline. Statistical significance reflects pairwise comparisons between each cancer cell line and MCF-10A at the corresponding time point (* *p* < 0.05, ** *p* < 0.01, and *** *p* < 0.001).

Intracellular pH (pH_i_) was quantified in MDA-MB-231 and MCF-7 breast cancer cells and in non-tumorigenic MCF-10A breast epithelial cells over a 6 h incubation period following treatment. Baseline pH_i_ values were consistent with the characteristic reversed pH gradient of cancer cells, with both MDA-MB-231 and MCF-7 cells exhibiting higher resting pH_i_ compared to MCF-10A cells. A time-dependent acidification response was observed in both cancer cell lines. MDA-MB-231 and MCF-7 cells maintained near-baseline pH_i_ during the initial 30 min, followed by a marked decrease beginning at 1 h and continuing through 3–6 h. This sustained intracellular acidification reflects a disruption of the cancer-associated alkaline pH_i_ phenotype. In contrast, MCF-10A cells showed minimal pH_i_ fluctuation across the entire time course, maintaining a stable intracellular pH despite treatment. Results are shown in Figure 5.

Collectively, these results demonstrate that CGA and CA lower intracellular pH in cancer cells significantly, while non-malignant epithelial cells are not affected. The collapse of pH_i_ in MDA-MB-231 and MCF-7 cells supports the hypothesis that CGA/CA disrupt cancer-specific pH homeostasis mechanisms while sparing normal cells.

### 3.5. OATP1B1 Expression Analysis by Immunofluorescence and Western Blotting

**Figure 6:**
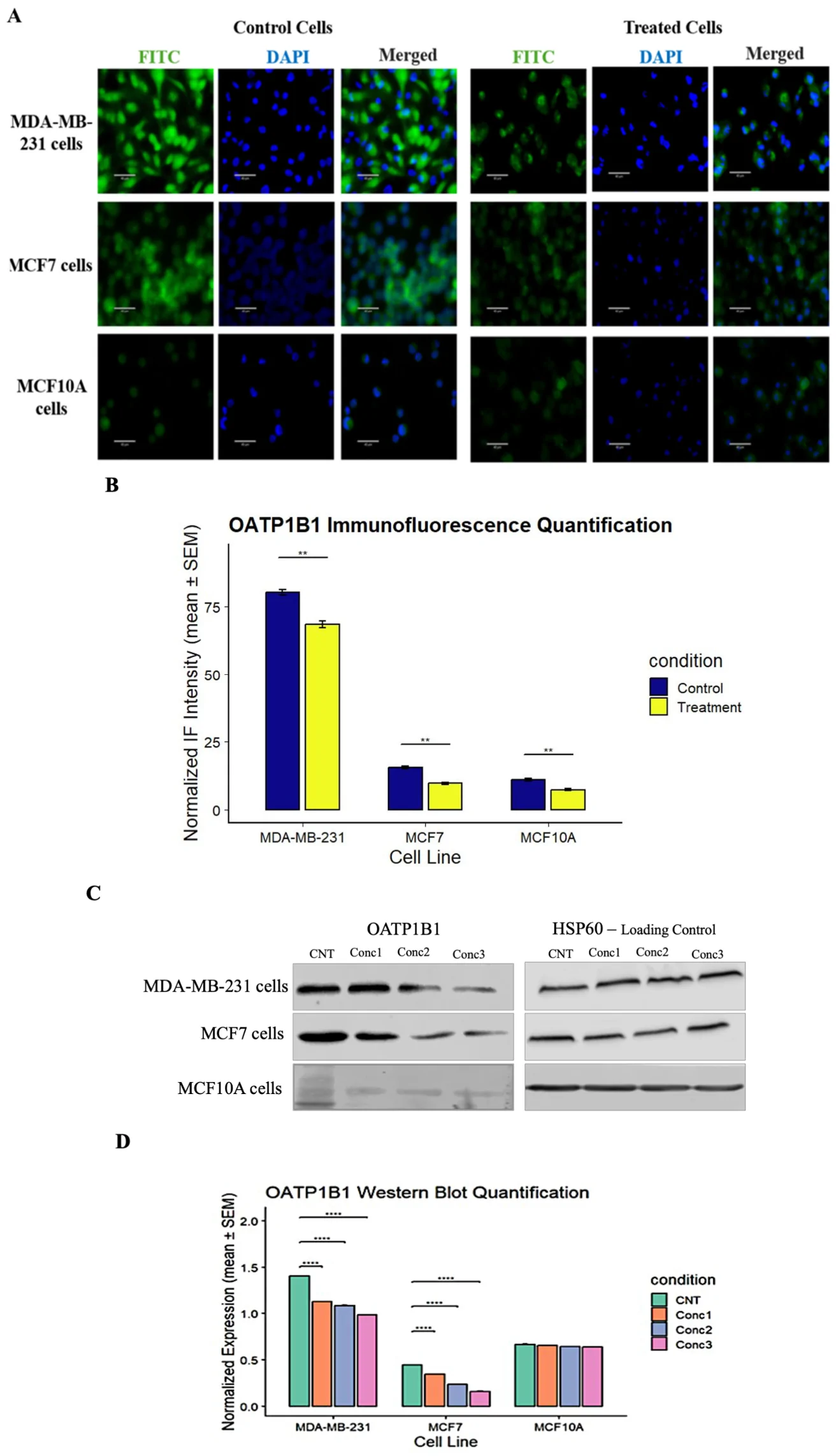
CGA:CA-induced modulation of OATP1B1 expression in breast cancer and non-cancer cells assessed by immunofluorescence (**A**,**C**) and Western blotting (**C**,**D**). Immunofluorescence imaging (**A**,**B**) shows strong OATP1B1-associated FITC signal in untreated MDA-MB-231 and MCF-7 breast cancer cells, whereas non-tumorigenic MCF-10A cells display minimal basal fluorescence. CGA:CA treatment markedly reduced OATP1B1 fluorescence intensity in both cancer cell lines, while MCF-10A cells exhibited little to no change relative to untreated controls. Western blot analysis (**C**,**D**) corroborates these findings: OATP1B1 protein levels were high in untreated MDA-MB-231 and MCF-7 cells and low in MCF-10A cells, with CGA:CA treatment significantly decreasing OATP1B1 band intensity in the cancer cell lines. HSP60 served as a loading control. The varied CGA and CA combinatorial concentrations used in these studies are described in the Section 2.2. Uncropped, original Western blots are located in Supplementary Figure S1. Quantification of fluorescence intensity and Western blot band density (right panels) confirmed these trends. Both analyses showed significant reductions in OATP1B1 expression in MDA-MB-231 and MCF-7 cells following CGA:CA treatment, whereas MCF-10A cells showed no statistically significant change. Data represent mean ± SEM from biological replicates. Statistical significance was determined using appropriate tests as indicated (** *p* < 0.01, **** *p* < 0.0001).

OATP1B1 expression was assessed in MDA-MB-231 and MCF-7 breast cancer cells and in non-tumorigenic MCF-10A epithelial cells using immunofluorescence microscopy and Western blotting, followed by quantitative analysis with statistical evaluation. Image J (version 1.54g) was used to measure the Western blot densitometry and mean fluorescence intensity. Figure 6A shows fluorescence microscopy results. Under untreated conditions, both cancer cell lines exhibited strong OATP1B1-associated FITC fluorescence, with MDA-MB-231 cells showing the highest signal intensity. In contrast, MCF-10A cells displayed minimal basal fluorescence, consistent with their non-malignant phenotype. CGA:CA treatment resulted in a pronounced reduction in OATP1B1 fluorescence in both cancer cell lines. Treated MDA-MB-231 and MCF-7 cells showed visibly diminished FITC signal relative to their untreated controls, indicating downregulation of the transporter. MCF-10A cells exhibited little to no changes in fluorescence following treatment, reflecting their low baseline expression (Figure 6B). Western blot analysis (Figure 6B) corroborated the immunofluorescence findings. A strong OATP1B1 protein signal was detected in untreated MDA-MB-231 and MCF-7 cells, whereas MCF-10A cells showed weak expression. CGA:CA treatment selectively reduced OATP1B1 band intensity in both cancer cell lines, while MCF-10A levels remained largely unchanged. HSP60 served as a loading control; Figure 6D. Quantification of both fluorescence intensity and Western blot band density confirmed these trends (Figure 6B,D). For simplicity, treatment concentrations are abbreviated in Figure 6D because the CA concentration differed between cell types. As described in the Methods Section 2.2, for MDA-MB-231 cells, the treatment combinations consisted of 25 µg/mL CGA + 10 µM CA (Conc1), 50 µg/mL CGA + 20 µM CA (Conc2), and 100 µg/mL CGA + 30 µM CA (Conc3). MCF-7 and MCF-10A cells received the same treatments. However, these differed from MDA-MB-231 cell treatments. MCF-7 and MCF-10A cell treatments were as follows: Conc1 at 25 µg/mL CGA + 10 µM CA (same as MDA-MB-231 cells), Conc2 at 50 µg/mL CGA + 15 µM CA, (which differed in CA from MDA-MB-231 cells), and Conc3 at 100 µg/mL CGA + 20 µM CA (which differed in CA from the MDA-MB-231 cells). Statistical analysis demonstrated significant reductions in OATP1B1 expression in MDA-MB-231 and MCF-7 cells following CGA:CA treatment, whereas no statistically significant changes were observed in MCF-10A cells. These quantitative results reinforce that CGA:CA significantly downregulates OATP1B1 in breast cancer cells while sparing non-malignant epithelial cells.

### 3.6. GLUT1 and MCT1 Expression Analysis by Immunofluorescence and Western Blotting

**Figure 7:**
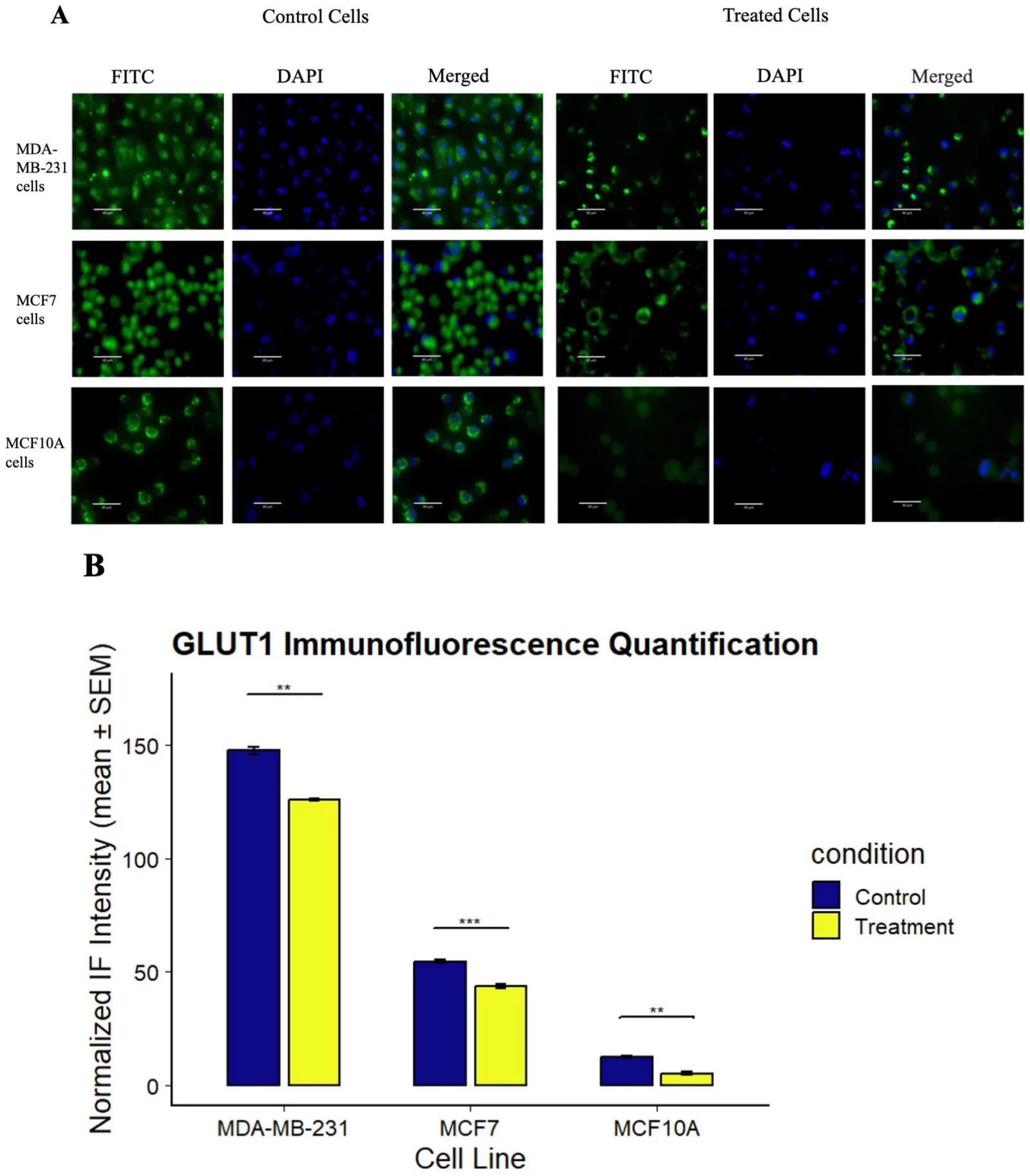

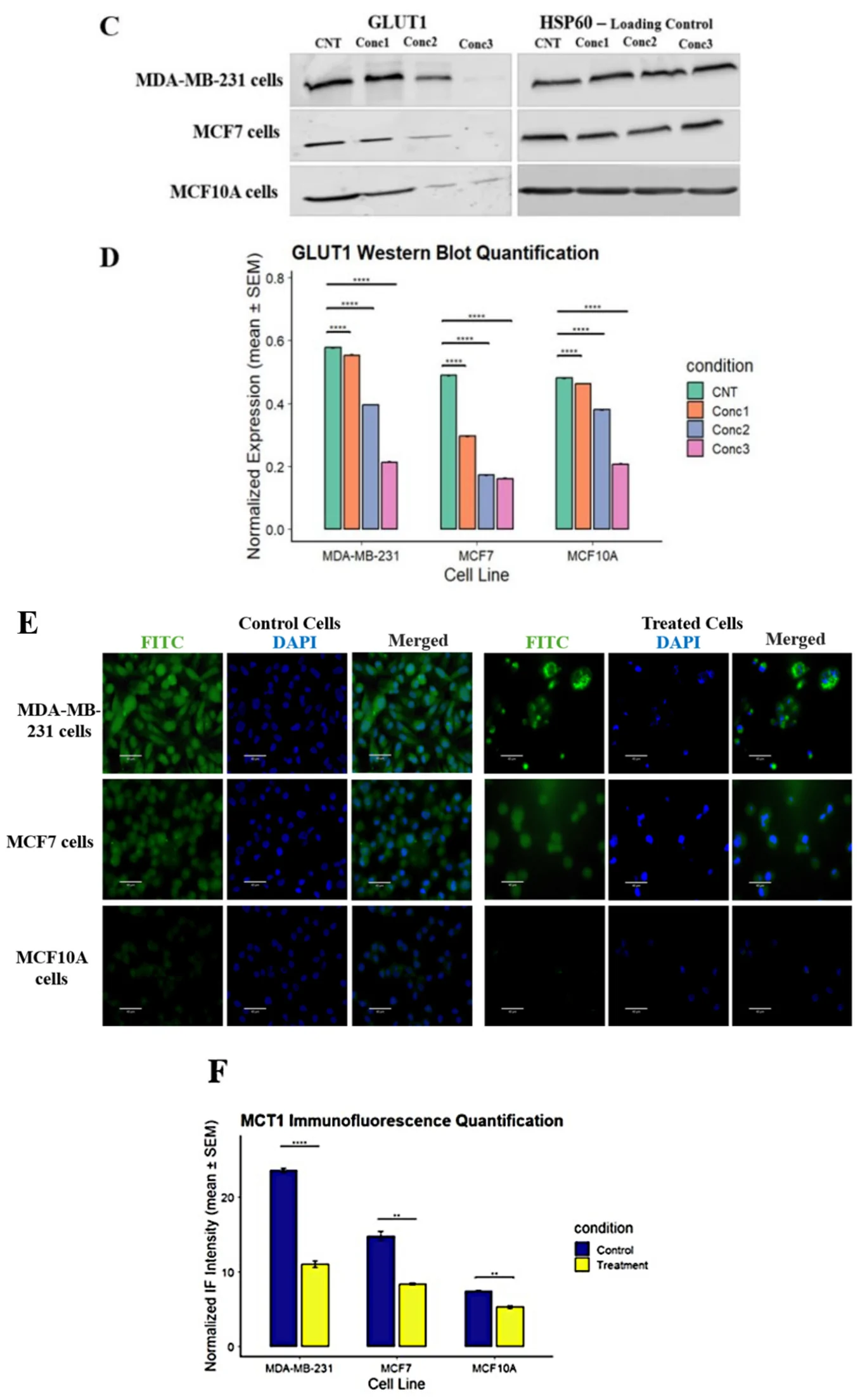

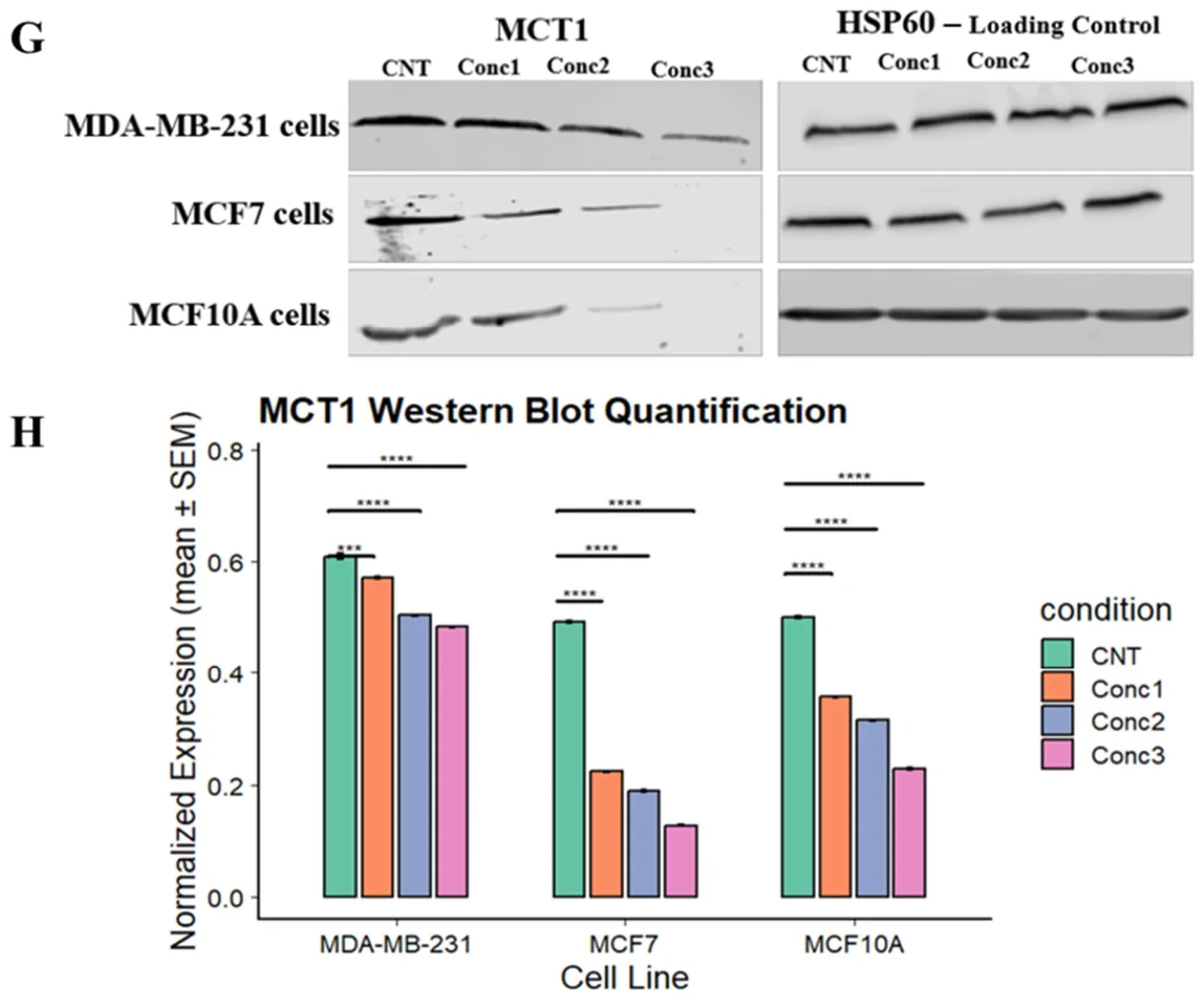
CGA:CA-induced downregulation of GLUT1 and MCT1 in breast cancer and non-cancer cells. Immunofluorescence imaging and quantification shows strong GLUT1 and MCT1 (**E**,**F**)-associated FITC signal in untreated MDA-MB-231 and MCF-7 breast cancer cells, whereas non-tumorigenic MCF-10A cells exhibit low basal fluorescence. Following CGA:CA treatment, while all cells displayed a significant reduction in FITC intensity for GLUT1(**A**,**B**) and MCT1 (**E**,**F**), indicating decreased transporter expression, the impact on MCF-10A cells is relative to untreated controls. For Western blot analysis, untreated MDA-MB-231 cells show high GLUT1 (**C**,**D**) and MCT1 (**G**,**H**) protein levels, whereas MCF-7 and MCF-10A cells express comparable, lower levels. CGA:CA treatment significantly reduces GLUT1 (**C**,**D**) and MCT1 (**G**,**H**) band intensities in the cancer cell lines, with less effect on MCF-10A. HSP60 served as a loading control. The varied CGA and CA combinatorial concentrations used in these studies were indicated in Section 2.2 and Section 2.7 of Methods. Uncropped blots are shown in Supplementary Figure S2. Data represent mean ± SEM from biological replicates, and statistical significance was determined using appropriate tests as indicated (** *p*< 0.01, *** *p*< 0.001, and **** *p* < 0.0001).

GLUT1 and MCT1 expression patterns were examined in MDA-MB-231 and MCF-7 breast cancer cells and in non-tumorigenic MCF-10A epithelial cells using immunofluorescence microscopy and Western blotting. Under untreated conditions, MDA-MB-231cancer cells exhibited strong FITC fluorescence for GLUT1 and MCT1, consistent with their elevated metabolic demands and reliance on glycolysis and lactate export. In contrast, MCF-10A cells showed markedly lower basal expression of both transporters, with MCF-7 cell fluorescence in the middle (Figure 7). CGA:CA treatment resulted in a significant reduction in GLUT1 and MCT1 fluorescence intensity in all three cell lines. Treated cancer cells displayed visibly diminished FITC signal relative to their untreated counterparts, indicating downregulation of both glucose uptake and lactate/H^+^ export pathways. MCF-10A cells showed a change in fluorescence following treatment. However, their basal fluorescence intensity was much lower to start with, reflecting their low baseline transporter expression and reduced metabolic dependence (Figure 7A,E). Western blot analysis corroborated the immunofluorescence findings. Strong GLUT1 and MCT1 protein bands were detected in untreated MDA-MB-231 cells, whereas MCF-7 and MCF-10A cells exhibited weaker, comparable expression under control conditions. CGA:CA treatment significantly decreased GLUT1 and MCT1 band intensities in both cancer cell lines, while MCF-10A levels remained significantly less affected for concentrations 1 and 2 (Figure 7C,D,G,H).

Quantification of fluorescence intensity and Western blot signal intensity further supported these observations. Both GLUT1 and MCT1 were significantly reduced in MDA-MB-231 and MCF-7 cells following CGA:CA exposure, whereas less significant changes were observed in MCF-10A cells compared to the cancer cell changes (Figure 7B,D,F,H). These results demonstrate that CGA:CA significantly suppresses key metabolic transporters in cancer cells, disrupting glucose uptake and lactate export pathways while having a comparatively reduced effect in the non-malignant epithelial cells. For simplicity, treatment concentrations are abbreviated in Figure 6D because the CA concentration differed between cell types. As described in the Methods Section 2.2, for MDA-MB-231 cells, the treatment combinations consisted of 25 µg/mL CGA + 10 µM CA (Conc1), 50 µg/mL CGA + 20 µM CA (Conc2), and 100 µg/mL CGA + 30 µM CA (Conc3). MCF-7 and MCF-10A cells received the same treatments. However, these differed from MDA-MB-231 cell treatments. MCF-7 and MCF-10A cell treatments were as follows: Conc1 at 25 µg/mL CGA + 10 µM CA (same as MDA-MB-231 cells), Conc2 at 50 µg/mL CGA + 15 µM CA (CA different from MDA-MB-231 cells), and Conc3 at 100 µg/mL CGA + 20 µM CA (CA different from the MDA-MB-231 cells).

## 4. Discussion

Breast cancer cells and other type of cancer cells rewire their metabolism to favor anaerobic glycolysis over oxidative phosphorylation, even in the presence of oxygen—a phenomenon known as the Warburg Effect [1,40]. To support this rapid metabolism, cancer cells overexpress specific transport proteins to import nutrients and export waste. A hallmark of breast cancer is the “reversed pH gradient,” where the intracellular environment is alkaline (pH_i_ ≥ 7.4) and the extracellular microenvironment is acidic (pH_e_ < 7.0) [40,41]. These metabolic and physical changes create a “fortress” that allows cancer cells to survive systemic therapies [1,40]. Because these metabolic adaptations extend beyond the tumor and impact systemic physiology, therapeutic strategies targeting them must be assessed in a manner that mirrors real-world clinical use [1,40]. Oral administration is particularly important because it exposes the compounds to the physiological processes of GIT absorption and pH variation, enzymatic metabolism, and hepatic first-pass metabolism, which determines their effectiveness at the tumor level [1,33,34,40]. For agents like chlorogenic acid (CGA) and cinnamaldehyde (CA), evaluating their oral stability and bioavailability ensures they can selectively target breast cancer’s metabolic vulnerabilities, such as its reversed pH gradient [30,32,34]. Using an in vitro model, the present study examined whether CGA and CA are pharmacokinetically stable and can effectively disrupt the biochemical features that sustain breast cancer cell survival. Specifically, this work evaluated pharmacokinetically relevant properties of CGA and CA—including their GIT-equivalent pH stability, hepatic first-pass metabolism, and intracellular uptake kinetics, as well as the ability of the combination (CGA:CA) to modulate cancer cell intracellular pH and expression of metabolic transport proteins.

The GIT-equivalent pH stability profiles of both CGA and CA, (as shown in Figure 2), reveal their chemical behavior during oral administration and their potential systemic availability. Both compounds possess ionizable functional groups whose protonation state is governed by the Henderson–Hasselbalch equation, which predicts the fraction of protonated versus deprotonated species at any given pH [42,43,44]. CGA, with carboxylic group pKa values typically 3.9, remains largely protonated and chemically stable under the acidic gastric pH of 1.2 tested. This finding is consistent with the Henderson–Hasselbalch relationship $\mathrm{pH}=\mathrm{pKa}+log\left(\frac{A^{-}}{HA}\right)$ prediction we reported previously that the ratio of deprotonated to protonated species will be extremely low [31]. This minimizes ionization-driven degradation and explains the high percentage of CGA remaining after incubation at this acidic gastric pH equivalent. Interestingly, CGA remains stable at the physiological pH of 7.4, confirming what we have reported previously [31]. This suggests that CGA may be stable at the near-neutral intestinal pH where maximum absorption may be occurring. However, at an alkaline pH of 9.0, the compound experienced accelerated degradation. Per the pH partition theory, we reported previously [31], CGA will become increasingly deprotonated in a higher pH milieu (like the colon), promoting intramolecular rearrangements, ester hydrolysis, and oxidative degradation. This is consistent with the accelerated loss of the parent compound we observed at pH 9.0.

CA, although structurally simpler and almost neutral in an aqueous solution with a pKa of about −4.4, exhibits a similar pH-dependent behavior to CGA—stable at both gastric and physiological pH but degraded in an alkaline environment. Its aldehyde moiety is susceptible to nucleophilic attack and base-catalyzed hydration under alkaline conditions, processes that are favored when the surrounding medium shifts above neutral pH [45]. This was further confirmed by our results. These chemical behaviors have meaningful biological and pharmacokinetic consequences. Because orally administered compounds must first transit the acidic stomach before entering the more neutral small intestinal epithelium, stability at low pH is essential for preserving the parent drug long enough to permit absorption [29,34,42]. The stability of both CGA and CA at pH 1.2 and 7.4 suggests they can survive gastric exposure and reach the enterocytes where absorption primarily occurs, thus enhancing their tissue penetration. Instability at the alkaline pH may be of less consequence to their absorption, as a large proportion of the parent molecules would have been absorbed in the small intestinal regions before reaching the colon. From an ADME perspective, pH-dependent degradation influences not only absorption but also distribution and metabolism. Thus, the results demonstrating stability in simulated gastric and intestinal environments support the feasibility of oral dosing [29,34,42].

The human liver microsomal (HLM) stability assay (Figure 3) provides a controlled system for evaluating whether a compound is susceptible to Phase I oxidative metabolism, particularly by cytochrome P450 (CYP) enzymes [32]. In this study, both CGA and CA showed minimal loss of the parent compound over 60 min in the presence or absence of NADPH, the essential cofactor required for CYP mediated oxido-reduction reactions. The absence of new metabolites in the reaction mixture indicates that neither CGA nor CA is a substrate for major hepatic CYP isoforms such as CYP3A4, CYP2D6, CYP2C9, or CYP1A2 [46]. This chemical stability is consistent with their structural features: the high polarity, the steric bulk of CGA aromatic ester scaffold, and the extensive hydrogen-bonding capacity render it poorly suited for productive engagement with hydrophobic CYP active sites [47,48]. In addition, CA’s aldehyde moiety is more prone to non-enzymatic hydration or oxidation than to CYP-catalyzed transformations [49,50]. The lack of metabolic turnover in HLMs therefore reflects both steric and electronic resistance to CYP-mediated oxidation [32]. That these compounds bypass extensive CYP metabolism suggests that they are less likely to undergo extensive first-pass metabolism following oral administration. This increases the probability that a larger fraction of the orally administered dose will reach systemic circulation intact, improving bioavailability. This stability is advantageous for CGA and CA, which must circulate systemically to reach their target tissues. Also, low CYP involvement reduces the risk of drug–drug interactions, since CYP inhibition or induction is a major source of clinical pharmacokinetic variability [51]. Compounds that bypass CYP metabolism are generally safer in polypharmacy settings [32,46]. This result also suggests that once the compounds are absorbed, these compounds may persist long enough to reach the tumor tissue and interact with cancer-associated transporters or disrupt intracellular pH regulation. Moreover, the minimal metabolism observed across two concentrations (10–20 µM for CA and 25–50 µg/mL for CGA) indicates that their stability is concentration-independent, reducing concerns about saturation kinetics or dose-dependent clearance. Overall, the HLM results demonstrate that CGA and CA are chemically and enzymatically stable in the hepatic microsomal environment, suggesting limited first-pass metabolism and favorable systemic availability [32,46].

The uptake kinetics of CGA and CA reveal clear differences in how malignant and non-malignant breast epithelial cells handle these compounds over time (Figure 4). Both molecules entered cells rapidly, reaching maximal intracellular levels within the first 30 min, consistent with transporter-facilitated uptake rather than slow passive diffusion [52]. However, MDA-MB-231 cells consistently accumulated and retained higher levels of both CGA and CA compared to MCF-10A cells, particularly at later time points. The sharper decline observed in MCF-10A cells indicates faster efflux, metabolism, or reduced intracellular retention. In contrast, the sustained levels in MDA-MB-231 cells suggest enhanced uptake capacity or slower clearance mechanisms characteristic of cancer cells [53]. These kinetic differences align with the broader metabolic and transporter phenotype of malignant cells [2,40,54]. MDA-MB-231 cells overexpress multiple solute carriers, including OATP family members, which can facilitate the uptake of polyphenolic and aromatic compounds such as CGA and CA [10,52,55]. The higher retention in cancer cells is also consistent with their altered bio-signaling and intracellular environment—basic intracellular pH that may cause their deprotonation and trapping, increased metabolic demand, and elevated transporter activity, as well as activated Akt signaling pathway and proteins downstream of the pathway—which may favor the sequestration or slower turnover of xenobiotics [2,6,12,31,40,56]. The early peak followed by a gradual decline suggests that CGA and CA rapidly engage with intracellular targets or undergo time-dependent metabolism, processes that may differ between malignant and non-malignant cells [2,6,12,31,40,56]. When taken together, these uptake patterns provide an important mechanistic foundation. The preferential accumulation of CGA and CA in cancer cells precedes the intracellular acidification (Figure 5), OATP1B1 suppression (Figure 6A–D), and downregulation of GLUT1 and MCT1 (Figure 7A–H) observed in the later experiments [2,6,12,31,40,56]. This temporal sequence supports a model in which early transporter-mediated uptake enables CGA and CA to reach effective intracellular concentrations that trigger metabolic stress and signaling disruption. The differential retention between MDA-MB-231 and MCF-10A cells also mirrors the selective vulnerability seen in downstream assays, reinforcing the idea that cancer-specific transporter expression and metabolic rewiring amplify the intracellular impact of CGA and CA. Therefore, the uptake kinetics highlight the importance of transporter biology in shaping the cellular response to CGA and CA and provide a mechanistic entry point for the multi-target metabolic and signaling disruptions observed throughout this current study and previous studies [2,6,8,9,10,12,13,40,52,56].

The intracellular pH (pH_i_) profiles observed (Figure 5) following CGA:CA treatment demonstrate a clear and selective disruption of the reversed pH gradient characteristic of breast cancer cells. Under basal conditions, both MDA-MB-231 and MCF77 cells maintain an abnormally alkaline cytosol (pH_i_ ≥ 7.4) relative to the extracellular space (pH_e_ ≤ 7.0), whereas nontumorigenic MCF-10A cells maintain a physiologically normal pH_i_ around 7.1 [5,27,40]. This reversed pH gradient is a hallmark of malignant transformation and arises from coordinated metabolic and transporter-driven processes [5,27,40]. Cancer cells upregulate proton extruding systems—including the Na^+^/H^+^ exchanger (NHE1), vacuolar H^+^ ATPase (V ATPase), monocarboxylate transporters (MCT1/MCT4), and carbonic anhydrase IX (CAIX)—to export protons and other metabolites like lactate generated by high glycolytic flux [5,6,12,13,27,40]. The result is a cytosol that remains alkaline enough to support proliferation, DNA synthesis, and resistance to apoptosis, while the extracellular space becomes acidic and promotes invasion, matrix degradation, and immune evasion [5,27,40]. Following CGA:CA exposure, both MDA-MB-231 and MCF77 cells exhibited a progressive decline in pH_i_ over the 6 h period, whereas MCF-10A cells maintained a stable intracellular pH. This selective acidification of cancer cells suggests that CGA and CA interfere with the biochemical systems that maintain the reversed pH gradient [5,6,12,13,27,40].

Several mechanisms may contribute to this effect. First, CGA and CA can act as weak acids capable of entering cells in their protonated form, via a carrier, and release protons intracellularly, thereby directly contributing to cytosolic acidification [3,42,57]. Second, both compounds may inhibit or compete with proton-coupled transporters such as MCT1 and MCT4, which are essential for exporting lactate and protons generated during aerobic glycolysis [58,59,60]. Inhibition of these transporters would trap acidic metabolites inside the cell, lowering pH_i_ [58,59,60]. Third, CGA has been reported to modulate carbonic anhydrase activity, and CA has been shown to interfere with mitochondrial function; either effect could reduce the cellular buffering capacity or proton consumption, further contributing to intracellular acidification [21,61,62]. Importantly, the phytochemicals can drive cytosolic acidification via negative regulation of the Akt pathway [14,15,31]. Akt regulates intracellular pH homeostasis by coordinating proton extrusion and metabolic flux [63,64]. Activated Akt directly phosphorylates and stimulates the Na^+^/H^+^ exchanger NHE1, enhancing proton efflux and intracellular alkalinization [64]. In parallel, Akt-driven glycolysis and lactate/proton export mechanisms maintain alkaline pH_i_ in cancer cells and inhibition of Akt disrupts these processes, resulting in intracellular proton accumulation and acidification [63]. The biological consequences of collapsing the reversed pH gradient are substantial. Cancer cells rely on an alkaline cytosol to maintain glycolytic enzyme activity, stabilize oncogenic signaling pathways, and prevent activation of the acid-sensitive apoptotic machinery [5,6,12,13,27,40]. Lowering pH_i_ disrupts these survival strategies by inhibiting glycolytic flux mediated by glucose transporters (GLUT), impairing ATP production, and destabilizing cytoskeletal and membrane dynamics [5,6,12,13,27,40]. Acidification also sensitizes cancer cells to oxidative stress and can trigger mitochondrial permeability transitions that promote apoptosis [2,21,26,40]. The stability of pH_i_ in the MCF-10A cells indicates that normal epithelial cells possess more robust homeostatic buffering systems and are less dependent on proton extruding transporters, making them less susceptible to CGA:CA-induced acid stress [19,20,21,52]. Overall, the intracellular pH data demonstrate that CGA and CA have a greater impact on disruption of the pH homeostasis of breast cancer cells than on non-malignant cells. By weakening the reversed pH gradient—a central metabolic adaptation that supports cancer survival, invasion, and treatment resistance—CGA:CA may sensitize malignant cells to metabolic stress and reduce their proliferative capacity [40,57]. These findings highlight intracellular pH modulation as a promising mechanism through which CGA and CA exert anticancer effects with greater impact.

The immunofluorescence and Western blot analyses demonstrate that OATP1B1 is differentially expressed across the three breast cell lines and is significantly downregulated following CGA:CA treatment (Figure 6). Under basal conditions, OATP1B1 expression is highest in the highly metastatic MDA-MB-231 cells, intermediate in low metastasis MCF-7 cells, and minimal in MCF-10A cells. This pattern is consistent with the metabolic phenotype of malignant cells, which frequently upregulate solute carrier transporters to support elevated nutrient uptake, xenobiotic handling, and metabolic flexibility [56]. The presence of OATP1B1 in all three cell lines—including the non-tumorigenic MCF-10A cells—confirms that the transporter is available to mediate uptake of exogenous organic anions such as CGA and CA [7,8,10,52,55]. Several lines of evidence from our study support the hypothesis that OATP1B1 functions as a carrier for CGA and CA [7,8,10,52,55]. First, both compounds possess structural features compatible with OATP1B1 substrates: CGA carries an aromatic catechol ring suitable for π–π interactions, a conjugated system for electron delocalization and a carboxyl group and polyol ring system that can H-bond with the residues in the OATP binding pocket, while CA contains an aromatic phenyl ring system that supports π–π stacking and van der Waals interactions and α-β unsaturated aldehyde for electronic stabilization and planarity, ensuring fitness into the OATP1B1 binding pocket—chemical motifs commonly recognized by OATP family transporters [52,55]. Second, the uptake kinetics demonstrate rapid intracellular accumulation of both compounds within the first 30 min, a time course consistent with facilitated transport rather than slow passive diffusion [53,65]. Third, the higher uptake of CGA and CA in MDA-MB-231 cells aligns with the elevated OATP1B1 expression in this line, suggesting their transporter-dependent entry [10,53,66]. Together, these observations strengthen the argument that OATP1B1 contributes to the cellular uptake of CGA and CA.

Following CGA:CA exposure, OATP1B1 expression decreases significantly in both cancer cell lines, and less so in the non-tumorigenic cells. This downregulation is consistent with a negative-feedback mechanism, in which transporter expression is suppressed after sufficient substrate has entered the cell [52]. Such feedback is well-documented for solute carriers involved in xenobiotic transport, where intracellular accumulation of substrate or substrate-induced signaling, such as a downregulated Akt signaling cascade, can trigger transporter internalization, degradation, or transcriptional repression [52,67]. In the context of CGA:CA treatment, the observed decline in OATP1B1 expression may reflect a cellular attempt to limit further influx of these compounds once intracellular concentrations reach a threshold, as the same transporter may drive their efflux [7,8,10,52,67]. The biological consequences of OATP1B1 downregulation are significant. Cancer cells rely on transporter-mediated uptake to sustain anabolic metabolism, maintain redox balance, and import substrates required for rapid proliferation [56,65]. Reducing OATP1B1 expression may impair the influx of organic anions, steroid conjugates, and metabolic intermediates, thereby weakening the metabolic adaptability of malignant cells [52]. This effect is particularly relevant given the intracellular pH data, which show that CGA:CA significantly acidifies cancer cells. Lower pH_i_ can destabilize transporter conformation, inhibit membrane trafficking, and suppress transcription of pH-sensitive genes, further contributing to OATP1B1 downregulation [3,41]. Furthermore, inhibition of the AKT pathway, which is the central regulator of expression, trafficking, and stability of membrane transporters, including several SLC family members, may be linked to OATP1B1 downregulation [31]. Inhibition of AKT decreases mTORC1 activity, thus limiting protein synthesis and accelerating turnover of high flux transporters such as OATP1B1 [31,68]. In this manner, CGA:CA may disrupt cancer cell homeostasis through different mechanisms, including initial transporter-mediated uptake followed by transporter suppression and intracellular acid stress, adding to the inhibitory effects on the AKT pathway [14].

As shown in Figure 7, treatment with CGA:CA resulted in a marked reduction in GLUT1 and MCT1 expression in cancer cells, as demonstrated by both immunofluorescence and Western blot analyses. In the IF images, control cells displayed strong green fluorescence corresponding to high transporter abundance, whereas treated cells showed visibly diminished signal intensity. This reduction was most pronounced in the highly glycolytic MDA-MB-231 cells, followed by MCF-7, with MCF-10A showing the smallest decrease. Western blot quantification confirmed these observations. GLUT1 and MCT1 protein levels decreased in a dose-dependent manner, with the highest treatment concentrations producing the most substantial suppression. The statistical significance across all comparisons (*p* < 0.01 to *p* < 0.0001) indicates that the downregulation is robust and reproducible. Together, these results, (Figure 6A–D and Figure 7A–G) demonstrate that CGA:CA treatment consistently suppresses two major metabolic transporters significantly in cancer cells. GLUT1 is the primary glucose transporter in many cancers and is essential for maintaining the Warburg phenotype, where cancer cells rely heavily on aerobic glycolysis [12,13]. Reduced GLUT1 expression limits glucose uptake, decreases glycolytic ATP production, reduces availability of glycolytic intermediates for biosynthesis, and increases metabolic stress [1]. Cancer cells, especially triple negative MDA-MB-231 cells, are highly dependent on GLUT1. Thus, its suppression directly compromises their metabolic flexibility and survival [12,54]. MCT1 is responsible for exporting lactate and protons produced during glycolysis to maintain intracellular pH [41,60]. Downregulation of MCT1 leads to intracellular lactate accumulation, intracellular acidification, inhibition of glycolytic enzymes, mitochondrial stress and activation of acid stress pathways [41,60,69,70]. This is consistent with the pH data we report (Figure 5), showing significant intracellular acidification in cancer cells following CGA:CA treatment. As cancer cells rely on a tightly coordinated system, combined GLUT1 and MCT1 suppression can collapse their metabolic homeostasis [1,40,65]. The metabolic transporter suppression by CGA:CA will shut down glycolytic flux, drive acid accumulation and attendant metabolic enzyme failure, with concomitant loss of cell viability and corresponding increase in cell death [41,56]. Akt is at the center of signaling pathways implicated in cancer cell survival, and we have previously reported the ability of CGA:CA to suppress AKT activation/phosphorylation and the proteins downstream of the Akt signaling [14,31]. AKT signaling is arguably a master regulator that can mechanistically promote GLUT1 membrane localization and maintain glycolytic metabolism; enhance MCT1 expression and regulate cancer cell pH dynamic; stabilize OATP1B1; and regulate xenobiotic transportation across the cancer cell membrane [68]. Therefore, CGA:CA-mediated AKT suppression can simultaneously reduce GLUT1, MCT1 and OATP1B1 expression, membrane incorporation and activity [10,68]. This can create a signaling cascade for metabolic collapse [14,15,31]. Furthermore, the acid stress induced by the natural products in cancer cells may in turn negatively affect the plasma membrane incorporation of GLUT1, inhibiting MCT1 trafficking, suppressing OATP1B1 transporter transcription and destabilizing membrane proteins [3,41]. Thus, intracellular acidification provides another layer to the regulation of metabolic transporter expression and activity [3,71].

In this study, we show that the combination of CGA and CA treatment disrupts cancer cell physiology through a coordinated sequence of pharmacokinetic, transporter-mediated, and metabolic events. Both compounds demonstrate sufficient GIT-equivalent pH stability and bypass CYP-mediated first-pass metabolism in HLM, supporting their potential to reach systemic circulation in active forms. Once available to cells, they exhibit efficient intracellular uptake, with OATP1B1 emerging as a key early mediator—consistent with CGA’s aromatic, conjugated and polyol-rich structure and CA’s planar, benzene ring and α,β-unsaturated aldehyde scaffold [52,55]. Following entry, CGA:CA induces a rapid intracellular acidification that significantly affects malignant cells, but not normal cells, indicating a disruption of pH homeostasis [3,41,71]. This acid stress coincides with a marked reduction in OATP1B1 expression, suggesting a negative feedback mechanism in which initial transporter-mediated uptake is followed by transporter suppression, limiting further influx [52,67]. This intracellular stress response extends to core metabolic transporters. GLUT1 and MCT1—critical regulators of glucose import and glycolytic flux, and lactate and proton export respectively—are significantly downregulated at both the protein and functional levels, collapsing the metabolic architecture that sustains the Warburg phenotype [12,59,60]. Reduced GLUT1 limits glucose availability, while diminished MCT1 impairs lactate and proton clearance and as a result, pH buffering is negatively affected, amplifying intracellular acidification and metabolic strain [12,40,41,60,62]. Together with our previous findings of Akt pathway suppression [14], these current findings support the hypothesis that CGA:CA compromises cancer cell viability by simultaneously targeting transporter-mediated uptake, intracellular pH regulation, and metabolic signaling. Taken together, this integrated pharmacokinetic stability and rapid uptake, acid–base balance modulation, and metabolic transport protein disruption establish CGA:CA as a promising multi-target metabolic signaling modulator with strong anticancer potential and reduced bystander effects.

## 5. Study Limitations

Although CGA and CA demonstrated favorable pH stability, microsomal stability, and measurable intracellular uptake in our in vitro models, these parameters alone do not fully define pharmacokinetic behavior in vivo. Critical determinants of drug disposition—such as membrane permeability, plasma protein binding, bioavailability, systemic clearance, and tissue distribution—were not evaluated in the present study. Accordingly, the term “pharmacokinetically favorable” constrained the limited scope of the biochemical and cellular assays performed. An ongoing study performed in an in vivo xenograft mouse study in our laboratory will directly assess several of these pharmacokinetic considerations, including compound exposure, tissue distribution, biological retention, and toxicological impact in a physiological context. These forthcoming data will help determine whether the stability and uptake characteristics observed in vitro translate to meaningful pharmacokinetic advantages in vivo.

A second limitation relates to our interpretation of CGA:CA-mediated disruption of the reversed pH gradient. While we quantified intracellular pH changes, extracellular pH was not directly measured. Because the reversed pH gradient in cancer involves both an abnormally alkaline intracellular environment and an acidified extracellular milieu, full characterization of the gradient collapse requires simultaneous assessment of both compartments. Although we observed consistent media color changes in CGA:CA-treated cancer cells (qualitatively suggesting increased acidification of the surrounding microenvironment), such observations cannot substitute direct extracellular pH measurements. Therefore, our conclusions regarding disruption of the reversed pH gradient are based primarily on intracellular pH modulation. Future studies, including our ongoing xenograft work, will incorporate dual intracellular–extracellular pH measurements to more comprehensively evaluate pH dynamics under physiological conditions.

Beyond the pharmacokinetic and pH-gradient considerations described above, other additional limitations need to be acknowledged. First, all mechanistic experiments were performed in vitro, which does not fully recapitulate the complexity of the tumor microenvironment, including stromal interactions, immune modulation, vascular perfusion, and systemic clearance. These physiological factors may influence CGA and CA disposition and biological activity in vivo. Thus, the current findings should be interpreted within the constraints of cell culture systems used. Second, the study evaluated only two breast cancer cell lines and one non-malignant epithelial line. While these models capture key malignant and non-malignant phenotypes, they do not encompass the full heterogeneity of breast cancer. Additional subtypes and genetically diverse models may reveal context-dependent differences in CGA and CA uptake, pH modulation, and metabolic stability. Finally, the concentrations used in vitro may not directly reflect physiologically achievable exposure levels following oral or systemic administration. Comprehensive pharmacokinetic studies, including those underway in our ongoing study using a xenograft model, will be required to determine clinically relevant dosing ranges, and to assess potential toxicological effects beyond viability-based endpoints.

### Conclusions

This investigation shows that combinatorial treatment with CGA and CA in vitro demonstrates a coordinated anticancer mechanism in which the compounds survive first-pass metabolism, enter cells through transporter-mediated uptake, and subsequently trigger intracellular acidification that suppresses OATP1B1, GLUT1, and MCT1 expression. Combining the findings of this study with our previous demonstration that CGA:CA negatively regulates the Akt pathway, characterized by inducing apoptosis and suppressing breast cancer cell invasion and migration [14,23,31], our results suggest that a combination of chlorogenic acid and cinnamaldehyde destroy cancer cell homeostasis through multi-target mechanisms with low side-effects in normal cells, increasing the rationale for exploring these natural products as viable anticancer therapies.

## Data Availability

Data is available upon reasonable request to the corresponding author.

## Supplementary Materials

The following supporting information can be downloaded at: https://www.mdpi.com/article/10.3390/molecules31162939/s1, Figure S1. (A) OATP1B1 blots—MW 76 kDa and (B) HSP60 blots—MW ~60 kDa; Figure S2. (A) Glut1 blots—45–60 kDa (average ~54 kDa) and (B) HSP60 blots – 60 kDa; Figure S3. (A) MCT1 blots—predicted MW 54 kDa (SDS-PAGE and Western blot analysis varies in molecular weights between 40 kDa and 45 kDa) and (B) HSP60 blots.
