# Supplementary material for: Combinatorial Treatment with Chlorogenic Acid and Cinnamaldehyde Disrupts Intracellular pH and Metabolic Transport in Breast Cancer Cells
Source: Molecules. 2026 Aug 21;31(16):2939. doi: 10.3390/molecules31162939 (PMC13515508; doi:10.3390/molecules31162939)
Supplement: Supplementary file 1 [file molecules-31-02939-s001.zip › molecules-4373824-supplementary.pdf]

Western blotting supplementary figures

A

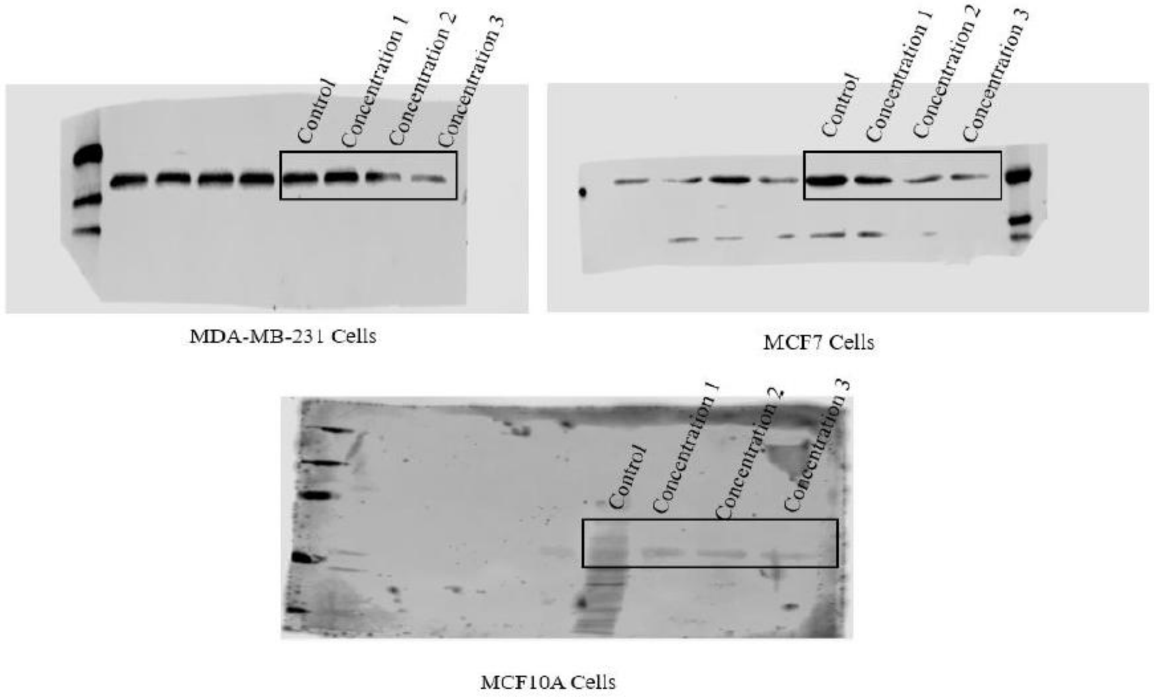

B

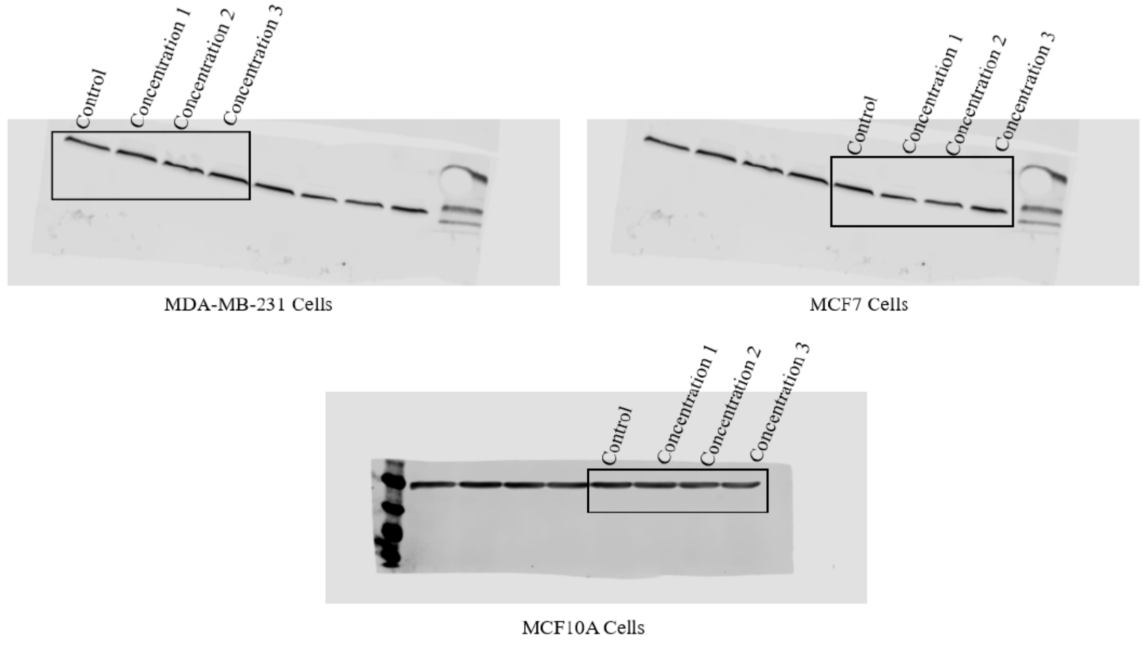

Figure S1. (A) OATP1B1 blots – MW 76 kDa and (B) HSP60 blots – MW ~60 kDa

A

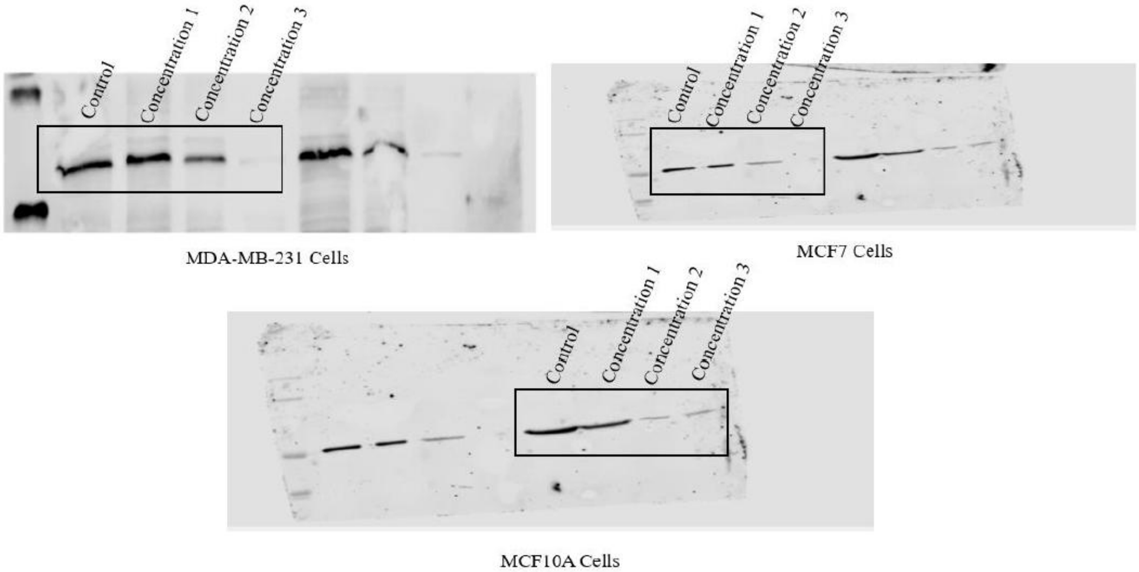

B

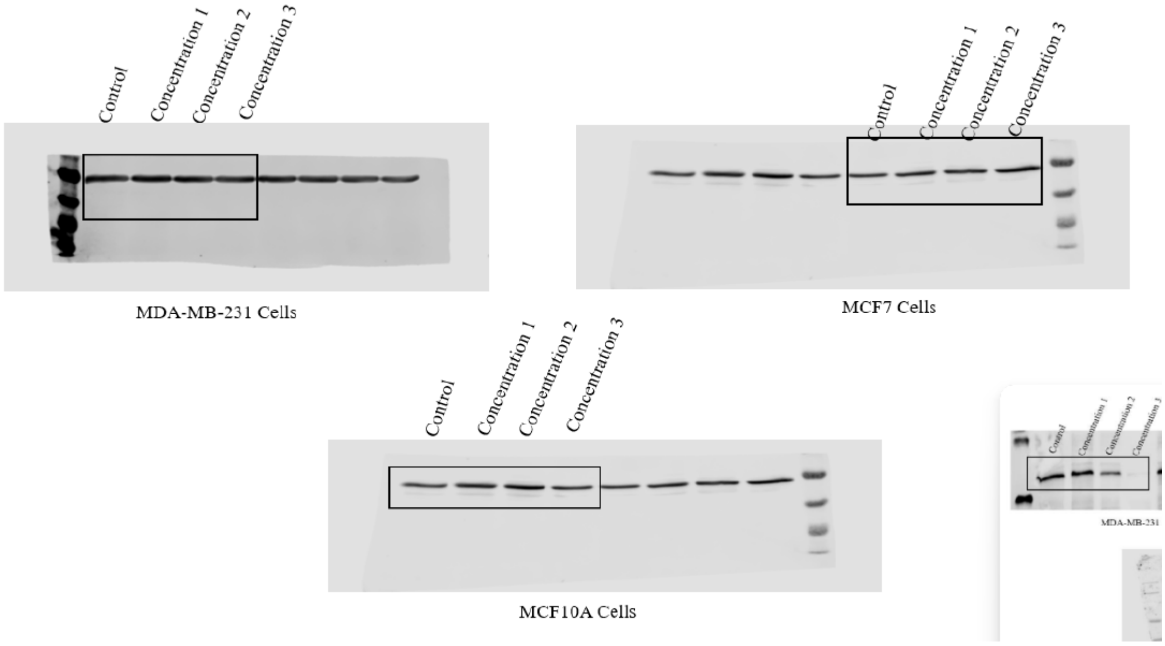

Figure S2. (A) Glut1 blots – 45-60 kDa (average ~54 kDa) and (B) HSP60 blots – 60 kDa

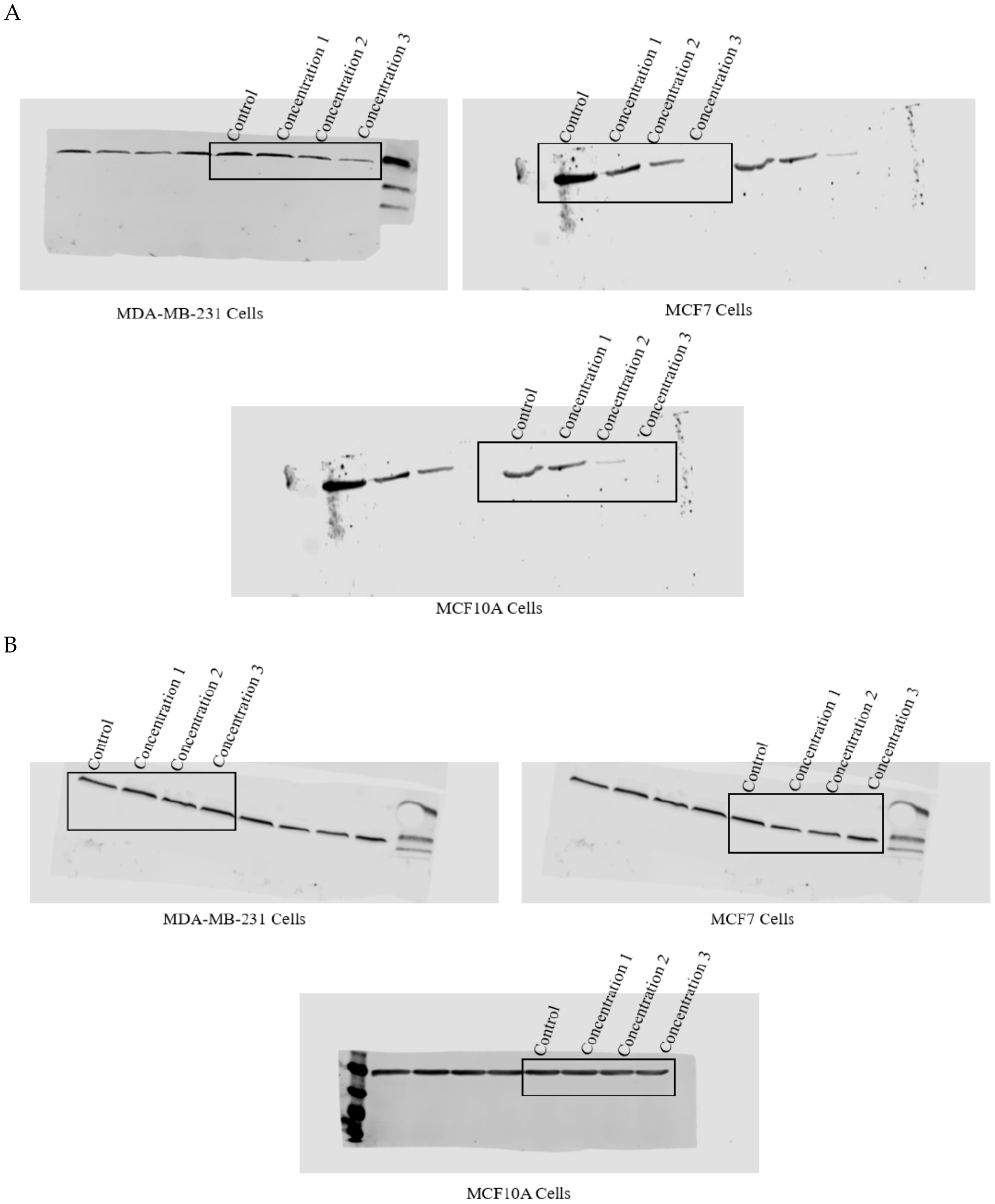

Figure S3. (A) MCT1 blots – predicted MW 54 kDa (SDS-PAGE and Western blot analysis varies in molecular weights between 40 kDa and 45 kDa) and (B) HSP60 blots
